# A Proresolving Peptide Nanotherapy for Site‐Specific Treatment of Inflammatory Bowel Disease by Regulating Proinflammatory Microenvironment and Gut Microbiota

**DOI:** 10.1002/advs.201900610

**Published:** 2019-08-01

**Authors:** Chenwen Li, Yang Zhao, Juan Cheng, Jiawei Guo, Qixiong Zhang, Xiangjun Zhang, Jiong Ren, Fengchao Wang, Jun Huang, Houyuan Hu, Ruibing Wang, Jianxiang Zhang

**Affiliations:** ^1^ Department of Pharmaceutics College of Pharmacy Third Military Medical University Chongqing 400038 China; ^2^ State Key Laboratory of Quality Research in Chinese Medicine Institute of Chinese Medical Sciences University of Macau Taipa Macau 999078 China; ^3^ State Key Laboratory of Trauma Burns and Combined Injury Institute of Combined Injury College of Preventive Medicine Third Military Medical University Chongqing 400038 China; ^4^ Institute for Molecular Engineering University of Chicago Chicago IL 60637 USA; ^5^ Department of Cardiology Southwest Hospital Third Military Medical University Chongqing 400038 China

**Keywords:** colitis targeting, gut microbiota, nanotherapy, proresolving peptide, reactive oxygen species

## Abstract

The incidence and prevalence of inflammatory bowel disease (IBD) increases steadily worldwide. There is an urgent need for effective and safe IBD therapies. Accelerated resolution of inflammation is a new strategy for the management of inflammatory diseases. For effective and safe IBD treatment, herein a smart nanotherapy (i.e. oxidation‐responsive nanoparticles containing a proresolving annexin A1‐mimetic peptide Ac2‐26, defined as AON) is developed, which can release packaged Ac2‐26, in response to highly expressed reactive oxygen species (ROS) at diseased sites. AON effectively protects Ac2‐26 from degradation in the enzyme‐rich environment of the gastrointestinal tract. By delivering this nanotherapy to the inflamed colons of mice with IBD, site‐specific release and accumulation of Ac2‐26 in response to high levels of ROS at the inflammatory sites are achieved. Mechanistically, the Ac2‐26‐containing, oxidation‐labile nanotherapy AON effectively decreases the expression of proinflammatory mediators, attenuates trafficking and infiltration of inflammatory cells, promotes efferocytosis of apoptotic neutrophils, and increases phenotypic switching of macrophages. Therapeutically, AON reduces symptoms of inflammation, accelerates intestinal mucosal wound healing, reshapes the gut microbiota composition, and increases short‐chain fatty acid production. Additionally, oral delivery of this nanomedicine shows excellent safety profile in a mouse model, conferring the confidence for further development of a targeted precision therapy for IBD and other inflammatory diseases.

## 1 Introduction

Inflammatory bowel disease (IBD) is a group of chronic disorders that cause prolonged inflammation of the gastrointestinal tract.^1^ Crohn's disease and ulcerative colitis are the principal types of IBD, affecting >3.6 million people in Europe and the United States.^2^ Although the etiology of IBD remains to be fully elucidated, conventional anti‐inflammatory drugs have been used for over four decades.^3^ These therapies, however, generally cause significant side effects. The enhanced understanding of molecular and cellular pathways involved in the pathogenesis of IBD has led to the development of biological therapies, such as antitumor necrosis factor and antiadhesion therapies.^4^ Unfortunately, nonspecific distribution of these biological agents often results in multiple adverse effects. Therefore, it is of high importance in developing specific, effective, and safe IBD therapies.

Inflammatory microenvironment plays an important role in the pathogenesis of a diverse array of inflammation‐associated diseases including IBD.^5^, ^6^ Throughout the entire process of the initiation, progression, and severity of IBD, there are complex molecular interactions and cellular communications among different proinflammatory cytokines/chemokines, oxidative mediators, inflammatory cells, and immune cells.^7^ In addition, the gut microbiota is closely related to the development of IBD.^8^ The inflammatory molecules, immune cells, and microbial community lead to the proinflammatory microenvironment in the intestine that causes IBD. Consequently, restoring the abnormal inflammatory environment of IBD represents an intriguing strategy toward effective therapy.

Recently, the resolution of inflammation has been considered as an active and tightly regulated process dominated by specific proresolving mediators, enabling suppression of inflammatory cytokine expression and clearance of apoptotic cells and microorganisms.^9^ Among different proresolving mediators, an endogenous protein annexin A1 can effectively treat inflammatory diseases.^10^ Ac2‐26 is an annexin A1 N‐terminal derived peptide with pharmacological activities of the whole protein.^11^ This peptide can inhibit various aspects of the inflammatory response, such as cell adhesion and transmigration, thereby reducing the infiltration of neutrophils and monocytes/macrophages.^12^ Ac2‐26 exhibits tissue protective actions in animal models of inflammation, such as peritonitis and atherosclerosis.^13^, ^14^ A recent study revealed the potential of Ac2‐26 for the treatment of colitis after intraperitoneal or intramucosal injection of Ac2‐26 in nanoparticles (NPs).^15^ Compared to the traditional therapies, such as mesalamine, aspirin, and dexamethasone, treatment with Ac2‐26 has few side effects due to its ability to mimic or induce natural pathways mediating the resolution of inflammation. On the other hand, patient‐friendly oral delivery is highly preferred for treatment of gastrointestinal and chronic diseases, owing to its convenience, high patient compliance, desirable cost‐effectiveness, and good safety profile.^16^, ^17^, ^18^, ^19^, ^20^, ^21^ However, there are still tremendous challenges in the design and development of translational nanovehicles for oral delivery of macromolecular therapeutics,^16^, ^22^, ^23^, ^24^, ^25^ although different nanotherapies have been developed for targeted treatment of IBD.^26^, ^27^, ^28^ While currently developed NPs are able to protect the loaded biologic drugs from hydrolysis in the gut, low bioavailability is generally observed due to insufficient and uncontrolled release at diseased sites, thereby leading to undesirable efficacies. Thus far, few nanotherapies derived from macromolecular drugs are in clinical development or on the market.^26^

As well documented, oxidative stress, characterized by overproduced reactive oxygen species (ROS), is closely related to the pathogenesis of IBD.^29^ Consequently, our hypothesis is that ROS‐responsive NPs can serve as triggerable vehicles for oral delivery of peptide therapeutics. Also, we assume that nanotherapy‐mediated simultaneous normalization of the proinflammatory microenvironment and dysbiosis in the intestine is an effective approach for the treatment of IBD. Based on our existing expertise in ROS‐responsive nanovehicles and the potency of Ac2‐26,^30^, ^31^, ^32^, ^33^, ^34^ we developed an Ac2‐26 peptide‐containing nanotherapy for site‐specific delivery to the inflamed colon in IBD. Both in vitro and in vivo studies were conducted to probe the mechanisms underlying therapeutic effects of this Ac2‐26‐derived proresolving nanotherapy.

## 2 Results

### 2.1 In Vivo Therapeutic Effects of Ac2‐26 in Mice with Dextran Sulfate Sodium‐Induced Colitis

First, in vivo efficacy of free Ac2‐26 was investigated in mice with dextran sulfate sodium (DSS)‐induced colitis. Treatment was conducted by daily oral administration at 250 µg kg^−1^ for 7 days. We found that the body weights of all DSS‐treated mice gradually decreased, regardless of Ac2‐26 treatment (Figure S1A, Supporting Information). Consistently, Ac2‐26‐treated colitis mice showed significant increase in disease activity index (DAI), fecal bleeding index, stool consistency index, and considerable decrease in colon length compared to those of normal mice (Figure S1B–E, Supporting Information). Furthermore, the manifestation of DSS‐induced inflammation in the colon was observed by miniendoscopy. DSS‐treated mice displayed clear symptoms of inflammation with significant ulcers and granular mucosal surface (Figure S1F, Supporting Information), and treatment with Ac2‐26 showed no beneficial effects. Also, inflammatory cytokines such as tumor necrosis factor (TNF)‐α and interleukin (IL)‐1β as well as oxidative stress‐related mediators including malondialdehyde (MDA) and hydrogen peroxide (H_2_O_2_) were significantly upregulated in the colon tissues isolated from DSS‐treated mice as compared to those of healthy mice (Figure S1G–J, Supporting Information). Of note, only the level of TNF‐α was significantly reduced after Ac2‐26 treatment, and no significant differences in the levels of IL‐1β, MDA, and H_2_O_2_ were found between colitis mice and diseased mice treated with Ac2‐26.

**Figure 1:**
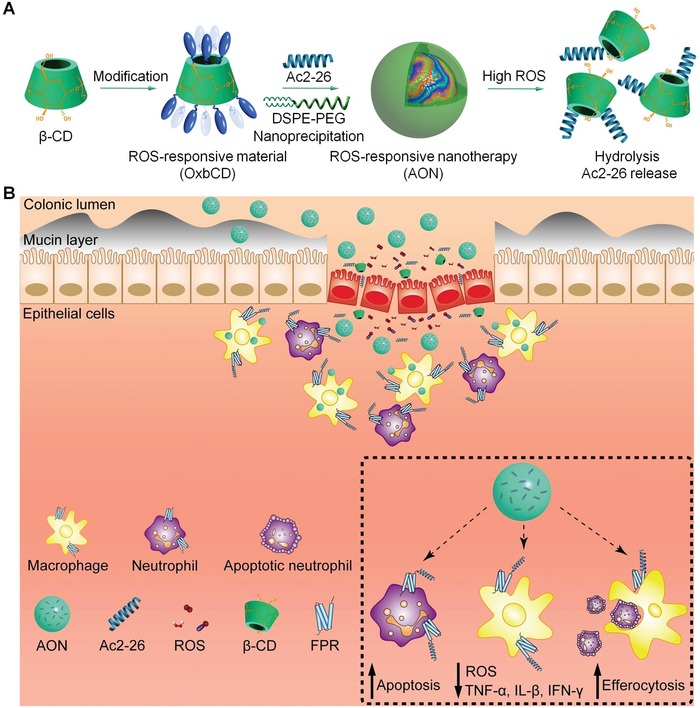
Schematic illustration of engineering of a ROS‐responsive peptide nanotherapy and targeted treatment of colitis. A) Development of a ROS‐responsive nanotherapy (AON) based on an inflammation‐resolving peptide Ac2‐26 derived from an endogenous proresolving protein annexin A1. A ROS‐responsive material (OxbCD) is synthesized by chemical functionalization of β‐cyclodextrin (β‐CD), while AON is prepared by a nanoprecipitation method. In the presence of high levels of ROS, hydrolysis of the OxbCD nanovehicle leads to release of Ac2‐26. B) A sketch showing targeted therapy of colitis by AON. AON can target the inflamed colon through the destructed intestinal barrier and the intrinsic adhesive capability of the nanocarrier. The packaged Ac2‐26 is released in response to high levels of ROS in microenvironment of the diseased colon, which then exerts its multiple biological activities via formyl peptide receptor (FPR)‐mediated signaling pathways.

Calculation of the organ index revealed significant splenomegaly and renomegaly in mice with colitis (Figure S2A, Supporting Information). Hematologic analyses indicated massive expansion of leukocytes as well as low levels of red blood cells and hemoglobin in the blood (Figure S2B–D, Supporting Information). Abnormally increased levels of alanine aminotransferase (ALT) and aspartate aminotransferase (AST) were also detected in diseased mice (Figure S2E,F, Supporting Information). With the exception of AST, treatment with free Ac2‐26 peptide showed very limited effects. Because Ac2‐26 is a peptide with 25 amino acids that is labile to hydrolysis in the harsh environment of the gastrointestinal tract after oral administration, we hypothesize encapsulation of Ac2‐26 into a ROS‐responsive nanovehicle will improve its oral efficacies by enhancing peptide stability and by enabling precision delivery to diseased sites (**Figure** **1**).

### 2.2 Preparation and Characterization of Ac2‐26‐Loaded NPs

**Figure 2:**
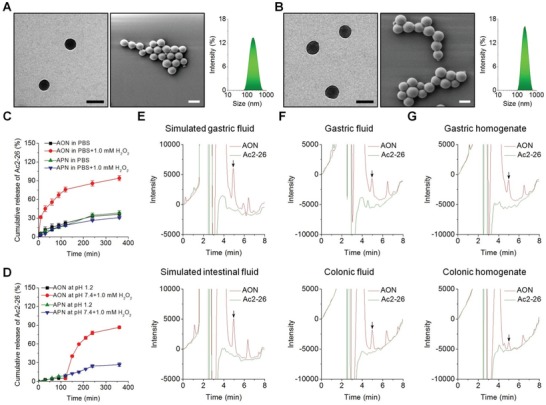
Characterization of Ac2‐26 nanotherapies. The TEM images (left), SEM micrographs (middle), and size distribution profiles (right) of A) AON and B) APN. Scale bars, 200 nm. C) In vitro release profiles of Ac2‐26 nanotherapies AON and APN in 0.01 m PBS at pH 7.4 with or without 1.0 × 10^−3^ m H_2_O_2_. D) Ac2‐26 release in buffers simulating the gastrointestinal pH conditions. Chemical stability of Ac2‐26 before and after loading into OxbCD NP and incubated in different solutions, including E) simulated gastric or intestinal fluids, F) gastric or colonic lavage fluids, and G) gastric or colonic homogenates. Data in (C) and (D) are presented as mean ± SE (*n* = 3).

A ROS‐responsive material (OxbCD) was synthesized by functionalizing β‐cyclodextrin (β‐CD) with an oxidation‐labile compound 4‐(hydroxymethyl)phenylboronic acid pinacol ester (PBAP) (Figure 1A and Figure S3A, Supporting Information).^32^ ^1^H nuclear magnetic resonance (NMR) spectroscopy and Fourier‐transform infrared spectroscopy demonstrated the successful synthesis of OxbCD (Figure S3B–C, Supporting Information). The ^1^H NMR spectrum revealed ≈7 PBAP moieties were linked to each β‐CD molecule. Ac2‐26 peptide was encapsulated into an OxbCD NP, using a nanoprecipitation/self‐assembly method, and this Ac2‐26‐containing, OxbCD‐derived nanotherapy was defined as AON. For AON, lecithin was used to afford it with an amphiphilic monolayer around the hydrophobic core of OxbCD, in which polyethylene glycol (PEG) chains were effectively anchored to provide desirable colloidal stability to this nanotherapy. Observation via transmission electron microscopy (TEM) and scanning electron microscopy (SEM) indicated that AON displayed a spherical shape (**Figure** **2**A, the left and middle panels). The mean diameter was 202 ± 4 nm, with a relatively narrow distribution of size (the right panel of Figure 2A; **Table** **1**). AON had a negative ζ‐potential of −37.4 ± 0.6 mV. For comparison, an FDA‐approved biodegradable material PLGA was used as a nonresponsive control material, and this Ac2‐26‐loaded PLGA NP was abbreviated as APN, which was also produced by the nanoprecipitation method (Figure 2B). AON and APN have comparable physicochemical properties (Table 1). The drug loading content of Ac2‐26 was 0.86 and 0.74 µg mg^−1^ for AON and APN, respectively.

**Table 1:** Physicochemical properties of Ac2‐26‐loaded NPs

	AON	APN
Diameter [nm]	202 ± 4	244 ± 3
ζ‐Potential [mV]	−37.4 ± 0.6	−34.6 ± 0.3
Entrapment efficiency [%]	42.8 ± 2.8	37.1 ± 2.6
Ac2‐26 loading content [µg mg^−1^]	0.86 ± 0.06	0.74 ± 0.05

### 2.3 Hydrolysis and In Vitro Release Profiles of Ac2‐26 Nanotherapies

AON displayed a rapid hydrolysis profile in 0.01 M PBS (pH 7.4) containing 1.0 × 10^−3^ m of H_2_O_2_, while almost no hydrolysis occurred after 6 h incubation in PBS alone (Figure S4A, Supporting Information). In the case of APN, we found slight hydrolysis in PBS with or without the presence of 1.0 × 10^−3^ m of H_2_O_2_ (Figure S4B, Supporting Information). Consistently, AON exhibited a rapid release profile in H_2_O_2_‐containing PBS (Figure 2C). By contrast, AON displayed slow Ac2‐26 release in different simulated gastrointestinal fluids without H_2_O_2_ (Figure S5, Supporting Information). As for APN, slow release was observed in PBS with H_2_O_2_.

We also examined the release of Ac2‐26 from two different nanotherapies in buffers mimicking gastrointestinal conditions (Figure 2D). For AON, only a small amount (≈5.0%) of Ac2‐26 was released after 2 h incubation at pH 1.2, thanks to the stability of the carrier material OxbCD and AON under the gastrointestinal condition (Figure S6, Supporting Information).^30^ In contrast, the release rate of Ac2‐26 remarkably increased in PBS (pH 7.4) containing 1.0 × 10^−3^ m H_2_O_2_. As for APN, similar slow release of Ac2‐26 was observed at both conditions. These results well agree with corresponding hydrolysis profiles (Figure S4, Supporting Information). Collectively, AON showed excellent ROS‐responsiveness in vitro.

### 2.4 The Stability of Ac2‐26 in AON

To ensure the peptide Ac2‐26 remains its therapeutic effects after oral administration, the AON should not only effectively deliver Ac2‐26 to the diseased site, but also maintain the structural integrity of Ac2‐26. To test whether Ac2‐26 in AON is stable in the harsh environment of the gastrointestinal tract, AON was incubated with simulated gastric or intestinal fluids for 2 h at 37 °C. The Ac2‐26 in AON was extracted for analysis by high‐performance liquid chromatography (HPLC). As expected, Ac2‐26 encapsulated in AON was clearly detected by HPLC (Figure 2E). Similar stabilities were found in gastric/colonic lavage fluids or gastric/colonic homogenates (Figure 2F,G). In contrast, free Ac2‐26 peptide completely hydrolyzed in all six solutions, consistent with the aforementioned results that orally administered Ac2‐26 was inactive in colitis mice (Figures S1 and S2, Supporting Information). Collectively, the results demonstrated that our AON can effectively protect Ac2‐26 from degradation under gastrointestinal conditions.

### 2.5 The Distribution of Orally Delivered AON in the Inflamed Colon

**Figure 3:**
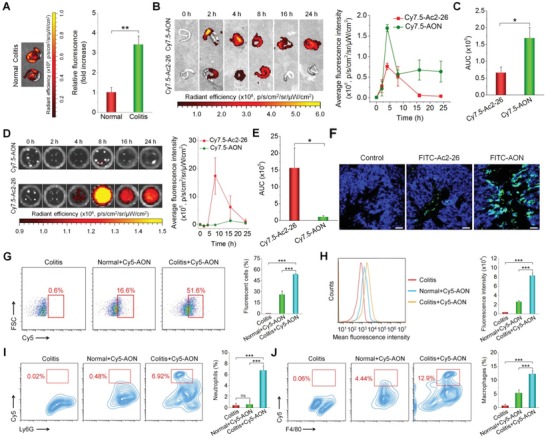
Selective accumulation of AON in the inflamed colons of mice with acute colitis. A) Representative ex vivo images (left) and quantitative analysis (right) illustrating Cy7.5‐AON distribution in the colons of mice with or without DSS‐induced acute colitis at 4 h after oral administration. The fluorescent signals of Cy7.5 revealed the accumulation of Cy7.5‐AON in the colons. B) Typical fluorescence images (left) and quantitative analysis (right) of the colonic tissues from colitis mice treated with free Cy7.5‐Ac2‐26 or Cy7.5‐AON. C) The area under the fluorescence intensity–time curve (AUC) of Cy7.5 fluorescence in colonic tissues after treatment with Cy7.5‐Ac2‐26 or Cy7.5‐AON. D) Fluorescence images (left) and quantitative analysis (right) of blood samples from colitis mice treated with free Cy7.5‐Ac2‐26 (top panel) or Cy7.5‐AON (bottom panel). E) The AUC value of Cy7.5 fluorescence of blood samples after treatment with Cy7.5‐Ac2‐26 or Cy7.5‐AON. F) Fluorescence images of cryosections of colonic tissues from mice with DSS‐induced acute colitis at 4 h after treatment with FITC‐Ac2‐26 or FITC‐AON. The green fluorescence indicates the presence of FITC‐labeled Ac2‐26, while the blue fluorescence reveals DAPI‐labeled nuclei. Scale bars, 100 µm. Representative flow cytometry plots (left) and quantitative analysis (right) indicating G) the percentage of Cy5‐AON‐containing cells and H) total fluorescence intensity of cells with Cy5‐AON. In both cases, cells were isolated from the healthy or inflamed colon tissues. The colitis group represents cells from diseased mice without treatment with Cy5‐AON. Distribution of Cy5‐AON in I) neutrophils and J) macrophages. The left panels show representative flow cytometry contour plots, while the right panels indicate corresponding quantitative data. All quantitative data are presented as mean ± SE (*n* = 3). *p < 0.05, **p < 0.01, ***p < 0.001; ns, no significance.

Effective delivery of AON to the diseased sites is a prerequisite for targeted therapy. We first examined the accumulation of AON at the injury sites of the inflamed colon, using AON loaded with Cy7.5‐labeled Ac2‐26 (abbreviated as Cy7.5‐AON). Colitis in mice was induced by oral administration of DSS in drinking water.^35^ At 4 h after oral delivery, ex vivo imaging showed significantly higher fluorescent signals in colons from colitis mice compared to those of healthy mice (**Figure** **3**A, the left panel). The fluorescence intensity of colitis tissues was 3.4‐fold higher than that of normal tissues from healthy mice (Figure 3A, the right panel). This was also affirmed by quantification of the Ac2‐26 content in the colonic tissues by HPLC (Figure S7A, Supporting Information). In addition, Cy7.5‐AON exhibited significantly higher accumulation in the diseased colon, compared to Cy7.5‐APN (Figure S8, Supporting Information). This can be explained by the adhesive property of OxbCD, as demonstrated in our previous studies.^31^, ^36^

We then evaluated the colonic accumulation of Cy7.5‐AON in mice with DSS‐induced colitis after oral administration. Compared to free Cy7.5‐labeled Ac2‐26 peptide, Cy7.5‐AON generally exhibited higher fluorescent signals (Figure 3B). In particular, Cy7.5‐AON showed a significant higher retention in the diseased colon. To quantify the degree of colonic targeting, we calculated the area under the fluorescence intensity–time curve (AUC). It was found that the AUC of Cy7.5‐AON was 2.6‐fold of that of Cy7.5‐Ac2‐26 (Figure 3C). In line with this result, Cy7.5‐AON‐treated mice displayed extremely low fluorescence distribution in the blood, while considerably high fluorescence intensity was observed for the free Cy7.5‐Ac2‐26 group (Figure 3D). The AUC of the free Cy7.5‐Ac2‐26 group was 15.3 folds of that of the Cy7.5‐AON group (Figure 3E). Consistently, compared to Cy7.5‐AON, the Cy7.5‐Ac2‐26 group exhibited higher nonspecific fluorescence distribution in the intestine, mesenteric lymph nodes (MLNs), liver, spleen, and kidney (Figures S9 and S10, Supporting Information), which are relevant to absorption, metabolism, and excretion, respectively.

In view of the poor stability of Ac2‐26 in the gastrointestinal tract (Figure 2E–G), the quantitative data by ex vivo imaging only indicated the fluorescence intensities of Cy7.5‐conjugated amino acid residues after metabolism of Cy7.5‐Ac2‐26, which approximately revealed the relative accumulation of Cy7.5‐Ac2‐26 itself. Consequently, the levels of Ac2‐26 in the colon, blood, MLNs, liver, spleen, and kidney of colitis mice were also quantified by HPLC at 4 and 24 h after oral administration of free Ac2‐26 or AON. In this case, the amount of Ac2‐26 in colonic tissues of the free Ac2‐26 group was much lower than that of the AON group at 4 and 24 h (Figure S7B, Supporting Information). Notably, almost no Ac2‐26 was detected in the colon of the free peptide group at 24 h. For both groups, no intact peptide was detected in the blood, MLNs, and kidney at 4 and 24 h after oral administration. Additionally, only very low contents of Ac2‐26 were found in the spleen and liver (Figure S11, Supporting Information).

Also, the enhanced targeting of AON to the inflamed colon was demonstrated by fluorescence imaging of cryosections of colonic tissues. After 4 h oral administration in colitis mice, the fluorescein isothiocyanate‐labeled AON (FITC‐AON) treated group showed notably higher fluorescence compared to that of the FITC‐Ac2‐26 group (Figure 3F). Furthermore, cellular distribution of orally administered AON was examined by flow cytometry, using AON containing Cy5‐labeled Ac2‐26 (Cy5‐AON). We found that both fluorescence‐positive cells and total cellular fluorescence intensity of colonic tissues from colitis mice were significantly higher than those from healthy mice (Figure 3G,H), well consistent with ex vivo imaging experiments (Figure 3A). To further identify which cells were targeted by orally delivered AON, neutrophils and macrophages were examined by flow cytometry for their internalization of Cy5‐AON after 6 h oral administration, since they are major inflammatory cells involved in the pathogenesis of colitis. For colitis mice, ≈6.8% of CD11b^+^Ly6G^+^ neutrophils and 12.3% of CD11b^+^F4/80^+^ macrophages were Cy5‐AON positive (Figure 3I,J). By contrast, Cy5‐AON^+^ neutrophils and Cy5‐AON^+^ macrophages were only 0.4% and 5.4% in the control healthy mice, respectively. Also, we found that Cy5‐AON exhibited significantly higher internalization in neutrophils and macrophages than Cy5‐APN (Figure S12, Supporting Information), which is consistent with the remarkably high accumulation of Cy7.5‐AON as revealed by ex vivo imaging (Figure S8, Supporting Information).

Taken together, packaging Ac2‐26 into the ROS‐responsive nanocarrier ON can effectively enhance its accumulation in the inflamed colon, concomitantly reducing its nonspecific distribution in the blood and other major organs. Moreover, a considerable amount of site‐specifically delivered AON was localized in inflammatory cells such as neutrophils and macrophages.

### 2.6 Therapeutic Effects of AON on Acute Colitis

**Figure 4:**
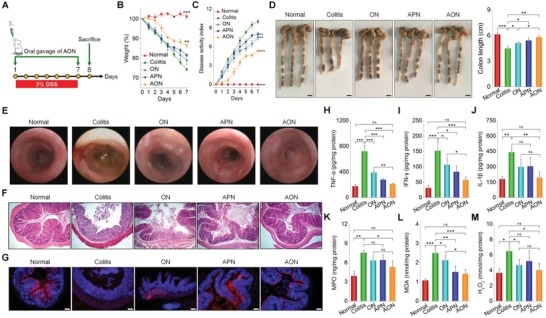
Therapeutic effects of AON on DSS‐induced acute colitis in mice. A) Schematic illustration of treatment regimens. B) The body weight of mice during a 7‐day treatment course. Data are normalized as the percentages of the body weight at day 0. **p < 0.01 and ***p < 0.001 versus the colitis group. C) Changes in disease activity index (DAI). **p < 0.01 and ***p < 0.001 versus the colitis group. D) Representative digital photos (left) and quantified lengths (right) of colonic tissues isolated from mice after 7 days of treatment. Scale bars, 5 mm. E) Representative miniendoscopic images of colons from mice at day 7 after different treatments. F) H&E‐stained histological sections of colonic tissues. G) Immunofluorescence analysis of colonic cryosections. Epithelial cells were stained with Cy3‐labeled anti‐cytokeratin 18 (CK18) antibody, and nuclei were stained with DAPI. Scale bars, 100 µm. The levels of H) TNF‐α, I) IFN‐γ, J) IL‐1β, K) MPO, L) MDA, and M) H_2_O_2_ in colonic tissues isolated from healthy or diseased mice treated with different formulations. After 7 days of treatment, homogenates of the colonic tissues were prepared, and the concentrations of various mediators were separately measured by the commercial kits. The total protein was measured by the BCA assay. All data are presented as mean ± SE (*n* = 6). For data in (D) and (H)–(M), *p < 0.05, **p < 0.01, ***p < 0.001; ns, no significance.

We further performed in vivo studies to evaluate the nanotherapy of the ROS‐responsive AON, using APN as a non‐ROS‐responsive control. Acute colitis in mice was induced by DSS, and simultaneously treated with different formulations (**Figure** **4**A). At day 7, AON‐treated mice showed less weight loss, lower DAI, and longer colon length, when compared to the model group treated with saline (Figure 4B–D). Also, AON effectively alleviated fecal bleeding and increased stool consistency (Figure S13A,B, Supporting Information). Additionally, miniendoscopic imaging showed significantly less inflammation in the colonic mucosa of AON‐treated mice, whereas marked symptoms of colitis such as ulcers and bleeding were observed in the colitis mice (Figure 4E). Therapy with ON and APN, however, offered lower efficacies (Figure 4B–E).

Examination on hematoxylin and eosin (H&E)‐stained sections showed that DSS‐induced colitis exhibited notably damages in colon structure with epithelial disruption, goblet cell depletion, and significant granulocyte infiltration (Figure 4F). While ON (the blank OxbCD NP) and APN treatment slightly attenuated colonic injuries, AON treatment cured the colitis, shown as nearly normal histological microstructure in AON‐treated mice. Further immunofluorescence analysis was performed to evaluate the integrity of colonic epithelium by examining the expression of cytokeratin 18 (CK18), an epithelial cell marker in the inflamed colon.^37^ Compared to other controls, the expression pattern of CK18 in AON‐treated mice was similar to that of normal mice (Figure 4G), indicating excellent colonic epithelial integrity after AON therapy.

Among all the treatments, AON significantly reduced the levels of important proinflammatory cytokines including TNF‐α, IFN‐γ, and IL‐1β, which showed no statistical differences compared to those of the normal control (Figure 4H–J). In addition, the activity of myeloperoxidase (MPO), a peroxidase associated with the presence of neutrophils in the mucosa and submucosa of the inflamed tissues, significantly decreased after AON treatment (Figure 4K). The levels of MDA and H_2_O_2_, two representative markers related to oxidative stress, were greatly suppressed after treatment with AON (Figure 4L,M). After AON treatment, colitis‐mediated renomegaly was almost completely reversed (Figure S13C, Supporting Information). Likewise, AON treatment completely restored the number of white blood cells and red blood cells as well as the level of hemoglobin and hematocrit (Figure S14A–D, Supporting Information). Consistent results were observed for ALT and AST, two biomarkers relevant to liver function (Figure S14E,F, Supporting Information). Treatment with ON and APN reduced the expression of these mediators to different degrees, while AON showed the most prominent effect. Histological analyses of H&E‐stained sections of the major organs and gastrointestinal tissues further showed no detectable injuries in AON‐treated groups (Figures S15 and S16, Supporting Information).

To further test the in vivo efficacy of AON, we investigated the effects of different orally administered formulations on the survival rate of mice with colitis induced by DSS. On day 12, all untreated animals died, while oral administration of Ac2‐26, ON, or APN slightly increased the survival rate to 20%, 40%, and 50%, respectively (Figure S17, Supporting Information). By contrast, AON treatment showed a survival rate of 70%. Taken together, our results demonstrated that AON achieved desirable efficacy in the treatment of acute colitis in mice, by effectively reducing inflammatory responses and suppressing oxidative stress.

### 2.7 Efficacy of AON Treatment on Chronic Colitis

**Figure 5:**
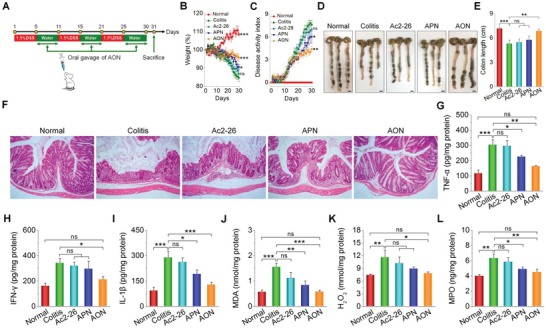
Therapeutic evaluations of AON in mice with DSS‐induced chronic colitis. A) The scheme of treatment procedures. Changes in the B) mouse body weight and C) DAI during 30 days of treatment. *p < 0.05, **p < 0.01, ***p < 0.001, and ns (no significance) versus the colitis group. Representative photos of D) colons, E) the colon length, and F) H&E‐stained histological sections of colonic tissues. Scale bars, 5 mm. The levels of representative inflammatory cytokines including G) TNF‐α, H) IFN‐γ, and I) IL‐1β in colonic tissues. The levels of oxidative mediators including J) MDA, K) H_2_O_2_, and L) MPO. For data in (E) and (G)–(L), *p < 0.05, **p < 0.01, ***p < 0.001; ns, no significance. All data are presented as mean ± SE (*n* = 6).

We next examined the in vivo efficacy of AON in treating chronic colitis, which was induced by repeated challenges with DSS (**Figure** **5**A). Mice treated with AON showed significantly improved weight loss, DAI, and colon length (Figure 5B–E). Histopathological evaluation revealed less colonic tissue damage (Figure 5F). In addition, therapy with AON strikingly decreased the expression of proinflammatory cytokines (TNF‐α, IFN‐γ, and IL‐1β) and oxidative stress‐related molecular mediators (MDA, H_2_O_2_, and MPO) in the colonic tissues (Figure 5G–L). Also, AON effectively mitigated the DSS‐induced renomegaly (Figure S18, Supporting Information). Consistent with the findings based on the acute colitis model, oral delivery of free Ac2‐26 showed no protective activities in this mouse model of chronic colitis. As for the nonresponsive control nanotherapy APN, it displayed considerably lower efficacy than that of AON. Meanwhile, long‐term oral administration of AON did not cause significant histopathological changes in H&E sections of typical major organs (including heart, liver, spleen, lung, kidney, and MLNs) and gastrointestinal tissues (Figures S19 and S20, Supporting Information). Both hematological parameters and biochemical markers relevant to hepatic/renal functions were in the normal ranges after AON treatment (Figure S21, Supporting Information).

In a separate study, in vivo efficacy of AON was compared with the mixture of the blank nanovehicle ON and Ac2‐26 in mice with DSS‐induced chronic colitis. To a certain degree, ON could reduce body weight loss, decrease DAI, maintain the colon length, and attenuate damage of colon tissues (Figure S22A–E, Supporting Information). Both inflammatory response and oxidative stress in colon tissues were also inhibited by treatment with ON, as implicated by decreased TNF‐α, IFN‐γ, IL‐1β, MDA, H_2_O_2_, and MPO (Figure S22F–K, Supporting Information). Nevertheless, therapy with the mixture of Ac2‐26 and ON afforded therapeutic effects comparable to those of ON alone. This is in line with the results that the activity of orally delivered Ac2‐26 was almost completely attenuated due to its hydrolysis (Figure S1, Supporting Information and Figure 5). It is worth noting that AON showed more desirable efficacy compared to ON alone or the mixture of Ac2‐26 and ON.

**Figure 6:**
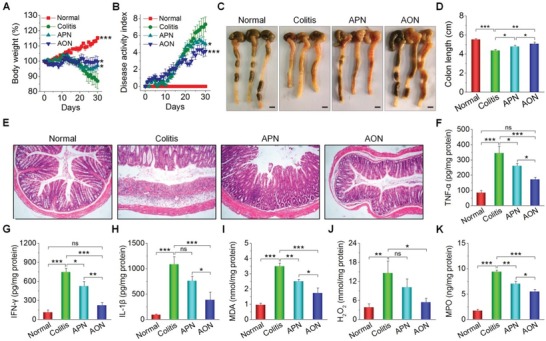
In vivo efficacy of AON in IL‐10^−/−^ mice with spontaneous colitis. Changes in the mouse A) body weight and B) DAI during 30 days of treatment. *p < 0.05, **p < 0.01, ***p < 0.001, and ns (no significance) versus the colitis group. Representative digital photos of C) colons, D) the colon length, and E) H&E‐stained histological sections of colonic tissues. Scale bars, 5 mm. The levels of F) TNF‐α, G) IFN‐γ, H) IL‐1β, I) MDA, J) H_2_O_2_, and K) MPO in colonic tissues. In all cases, healthy C57BL/6J mice were administered with saline in the normal group, while IL‐10^−/−^ mice were treated with saline, APN, and AON for the Colitis, APN, and AON groups, respectively. For data in (D) and (F)–(K), *p < 0.05, **p < 0.01, ***p < 0.001; ns, no significance. All data are presented as mean ± SE (*n* = 6).

Furthermore, the protective effects of AON treatment were examined in IL‐10‐deficient (IL‐10^−/−^) mice that can spontaneously develop colitis. Compared to wild‐type mice, IL‐10^−/−^ mice received saline showed gradually decreased body weight and increased DAI, particularly after 10 days (**Figure** **6**A,B), which were mitigated by treatment with either APN or AON. After 30 days of therapy, the significantly improved colon length and notably inhibited colon injuries were achieved in APN and AON groups (Figure 6C–E). The severity of inflammation and oxidative stress was also significantly decreased by two nanotherapies (Figure 6F–K). In all these cases, AON showed much better therapeutic effects compared to APN, which was further confirmed by quantification of representative hematological parameters (Figure S23A–D, Supporting Information). Of note, long‐term treatment with AON did not cause discernible adverse effects on major organs and gastrointestinal tissues in IL‐10^−/−^ mice (Figures S23E–H, and S24 and S25, Supporting Information). Collectively, these results demonstrated that AON was also effective for the treatment of chronic colitis.

### 2.8 The Anti‐Colitis Mechanisms of AON In Vitro and In Vivo

#### 2.8.1 In Vitro Antioxidation and Anti‐Inflammatory Activities of AON

**Figure 7:**
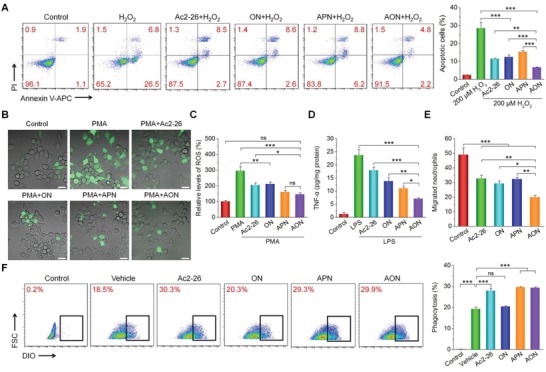
In vitro antioxidation and anti‐inflammatory activities of AON. A) H_2_O_2_‐induced apoptosis of macrophages shown by flow cytometry plots (left) and corresponding quantitative analysis (right). Before exposure to 200 × 10^−6^ m H_2_O_2_ for 12 h, macrophages were preincubated with different formulations for 4 h. B) Fluorescence microscopy images showing the intracellular generation of ROS in macrophages stimulated with 100 ng mL^−1^ of PMA and treated with different formulations for 4 h. DCFH‐DA was used as a fluorescent probe to stain intracellular ROS. Scale bars, 20 µm. C) The relative ROS levels in macrophages quantified by flow cytometry. Cells were treated with the same procedures as for fluorescence microscopy. D) The expression levels of TNF‐α in macrophages after different treatments. Macrophages were preincubated with different formulations for 6 h, and then exposed to 100 ng mL^−1^ of LPS for 12 h before quantification of TNF‐α. E) Quantitative results of migrated neutrophils in the lower chamber of the Transwell system after treatment with different formulations. F) Phagocytosis of apoptotic neutrophils by macrophages shown by flow cytometry (left) and corresponding quantitative analysis (right). Macrophages were exposed to vehicle, Ac2‐26, ON, APN, or AON for 1 h before the addition of labeled apoptotic neutrophils. In the control group, no apoptotic neutrophils were added. Data are presented as mean ± SE (*n* = 3). *p < 0.05, **p < 0.01, ***p < 0.001; ns, no significance.

We examined in vitro antioxidative stress and anti‐inflammatory activities of different Ac2‐26 formulations. While H_2_O_2_ caused significant apoptosis of mouse macrophage RAW264.7 cells (**Figure** **7**A), pretreatment with different formulations including free Ac2‐26, ON, APN, and AON significantly inhibited H_2_O_2_‐induced apoptosis. Among different formulations, AON was the most potent one that effectively suppressed intracellular ROS generation induced by phorbol 12‐myristate 13‐acetate (PMA), shown by both confocal microscopy and flow cytometry analyses (Figure 7B,C). It should be emphasized that treatment with AON more significantly inhibited PMA‐induced ROS generation, compared to the same dose of Ac2‐26/ON mixture (Figure S26A, Supporting Information). The data showed that Ac2‐26 attenuated oxidative stress‐induced cell apoptosis by eliminating intracellular ROS, and packaging into NPs (particularly the ROS‐responsive ON) further potentiated its antioxidant activity. Of note, ON exerted antiapoptotic activity comparable to that of Ac2‐26, which should be due to its H_2_O_2_‐scavenging capability (Figure 7A–C), agreeing with our previous finding.^30^

We then examined in vitro anti‐inflammatory activities of Ac2‐26 in different formulations. Compared to the normal RAW264.7 cells, lipopolysaccharide (LPS)‐treated cells expressed a significantly higher level of TNF‐α (Figure 7D), a proinflammatory cytokine that is mainly secreted by macrophages during the onset and progression of ulcerative colitis.^38^ Pretreatment with Ac2‐26, Ac2‐26/ON mixture, and different Ac2‐26‐containing NPs effectively reduced the TNF‐α levels in macrophages treated with LPS (Figure 7D and Figure S26B, Supporting Information). Again, AON was more effective than APN and Ac2‐26/ON mixture. The results demonstrated the anti‐inflammatory activity of Ac2‐26 nanotherapy in vitro.

#### 2.8.2 Migration and Efferocytosis of Neutrophils

Neutrophils play a crucial role in acute inflammation. They can be rapidly recruited to sites of infection or injury.^39^ However, excessive neutrophil recruitment generally leads to persisting tissue damage in many acute and chronic inflammatory diseases. We first examined how Ac2‐26 and its nanotherapies regulate the migration of neutrophils by a transwell migration assay. Compared to the untreated cells, neutrophils treated with Ac2‐26, ON, APN, and AON displayed significantly low migration (Figure S27, Supporting Information and Figure 7E). AON was the most effectively one in inhibiting neutrophil migration among different formulations.

Phagocytosis of apoptotic neutrophils by macrophages is a key step in the resolution of inflammation. Flow cytometric analysis suggested that pretreatment of macrophages with free Ac2‐26, APN, or AON resulted in a similar degree of increased phagocytosis of apoptotic neutrophils (Figure 7F). Notably, ON (i.e., the OxbCD NP alone without Ac2‐26) showed no inhibitory activity. This result suggested that the phagocytosis‐promoting function mainly driven by Ac2‐26 with a ROS‐independent manner. These results demonstrated that both Ac2‐26 and its nanotherapies can significantly suppress the migration of neutrophils and remarkably promote macrophage phagocytosis of apoptotic neutrophils.

#### 2.8.3 Formyl Peptide Receptor (FPR) Signaling‐Mediated Biological Activities of AON

**Figure 8:**
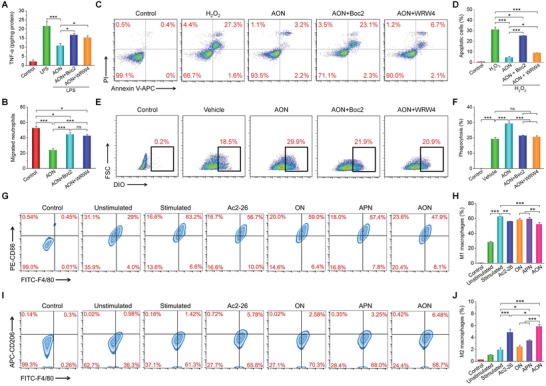
FPR signaling‐mediated anti‐inflammation and AON‐regulated macrophage polarization. A) The expression levels of TNF‐α after treatment with FPR inhibitors. Macrophages were pre‐incubated with AON, AON/Boc2 combination, or AON/WRW4 combination, followed by exposure to LPS for 12 h. B) Migration of neutrophils after treatment with AON in the absence or presence of Boc2 or WRW4. C,D) Flow cytometry analysis of macrophage apoptosis. Macrophages were preincubated with AON, AON/Boc2, or AON/WRW4 for 4 h, and then treated with H_2_O_2_ for 12 h. Cells in the control group were treated with fresh medium without H_2_O_2_. E,F) Phagocytosis of apoptotic neutrophils by macrophages shown by flow cytometry analysis. Macrophages were treated with vehicle, AON, AON/Boc2, or AON/WRW4 for 1 h before addition of labeled apoptotic neutrophils. In the control group, no apoptotic neutrophils were added. G–J) AON regulates phenotypic switching of macrophages in vitro. RAW264.7 macrophages were stimulated with 200 ng mL^−1^ LPS and 2.5 ng mL^−1^ IFN‐γ for 24 h, and then treated with various formulations for 8 h. Flow cytometric analysis illustrating the number of G,H) M1 (F4/80^+^CD86^+^) and I,J) M2 (F4/80^+^CD206^+^) macrophages. Data are presented as mean ± SE (*n* = 3). *p < 0.05, **p < 0.01, ***p < 0.001; ns, no significance.

FPR, a cell surface receptor of the proresolving peptide Ac2‐26, is expressed on macrophage surface. We examined whether the anti‐inflammatory effects of AON are attributed to the binding of Ac2‐26 to FPRs and subsequent signaling and activation of macrophages, using a nonselective FPR antagonist Boc2 and a selective FPR2 antagonist WRW4. Pretreatment of RAW264.7 cells with AON for 4 h significantly reduced the LPS‐induced expression of TNF‐α, and its levels were restored by either Boc2 or WRW4 treatment (**Figure** **8**A). Consistently, the antimigration effect of AON to neutrophils was blocked by Boc2 or WRW4 (Figure S28, Supporting Information and Figure 8B). In addition, Boc2 or WRW4 significantly reduced antiapoptotic and efferocytosis‐enhancing activities of AON in macrophages (Figure 8C–F), with Boc2 showed a stronger effect than that of WRW4. Together, these results demonstrated that the protective actions of AON were primarily through the signaling and activation of FPRs on macrophages.

#### 2.8.4 In Vitro Phenotypic Switching of Macrophages Regulated by AON

We further examined the effects of AON on macrophage polarization. For RAW264.7 macrophages, treatment with LPS and IFN‐γ induced a strong M1 response, as shown by the significantly increased number of CD86^+^ cells (Figure 8G,H). After incubation with free Ac2‐26 or AON, the expression of the M1 biomarker CD86 significantly decreased compared to that of the untreated control. AON treatment remarkably increased the expression of CD206, a biomarker of the M2 phenotype (Figure 8I,J). Both free Ac2‐26 and APN promoted macrophage polarization, but ON had no effects. These results suggested that AON promoted the macrophage phenotypic switching from an inflammatory M1 phenotype (F4/80^+^CD86^+^) to an alternatively activated M2 phenotype (F4/80^+^CD206^+^) that is beneficial for the repair of various types of organs.^40^

#### 2.8.5 AON Decreased the Recruitment of Inflammatory Cells In Vivo

**Figure 9:**
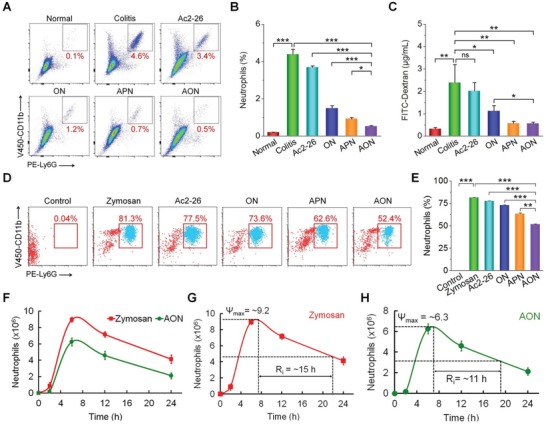
In vivo anti‐inflammation, epithelial wound healing, and resolving inflammation by Ac2‐26 nanotherapies. A,B) Flow cytometric analysis illustrating the percentages of neutrophils (CD11b^+^Ly6G^+^) in single‐cell suspensions derived from colonic tissues of mice with acute colitis after 7 days of treatment. C) The effect of Ac2‐26 nanotherapies on epithelial wound healing in vivo. Mice with DSS‐induced acute colitis were treated with PBS, Ac2‐26, ON, APN, or AON for 7 days. The barrier function was assessed at day 7. Each mouse orally received 12 mg FITC‐Dextran, and blood was sampled at 4 h after administration. The permeability of intestinal epithelium was evaluated by measuring the fluorescence intensities. D–H) Proresolving activity of Ac2‐26 nanotherapies in mice with peritonitis. Representative D) flow cytometry dot plots and E) quantitative analysis of peritoneal exudate cells. Neutrophils are indicated by CD11b^+^Ly6G^+^ cells. F) Time‐dependent changes of neutrophils in peritoneal exudates after zymosan‐challenged mice were treated with AON. Analysis of resolution indices for G) zymosan alone and H) AON. *Ψ*_max_, the maximal number of neutrophils; *R*_i_, the interval between the time point when neutrophils reach *Ψ*_max_ and the time point corresponding to ≈50% neutrophil reduction. Data are presented as mean ± SE (*n* = 3, B–E; *n* = 6, F–H). *p < 0.05, **p < 0.01, ***p < 0.001; ns, no significance.

Because neutrophils and macrophages are crucial innate immune cells in inflammation such as IBD,^41^ we tested the effects of AON in the recruitment of neutrophils and macrophages in vivo. After 7 days of treatment of DSS‐induced colitis with AON, the number of CD11b^+^Ly6G^+^ neutrophils and CD11b^+^F4/80^+^ macrophages recruited to the inflamed colons significantly decreased compared to the untreated group (**Figure** **9**A,B and Figure S29, Supporting Information). Among different treatments, AON was more efficacious than free Ac2‐26 or APN. The nanocarrier ON also showed regulatory effects. This implicated that elimination of H_2_O_2_ alone was able to decrease the recruitment of inflammatory cells.

#### 2.8.6 Oral Administration of AON Accelerated Intestinal Mucosal Wound Healing

Intestinal wound healing is closely involved in the pathogenesis of various gut diseases.^42^ We investigated the efficacy of AON treatment on wound healing of the intestinal mucosa, using FITC‐labeled dextran (FITC‐Dextran) as a fluorescent probe. After oral administration of FITC‐Dextran in mice with acute colitis, the untreated model group showed a significantly higher level of FITC‐Dextran in serum than groups treated with different nanotherapies (Figure 9C). This finding is well consistent with the result based on histological and immunofluorescence analyses (Figure 4F,G). Particularly, both AON and APN promoted intestinal epithelial wound repair, whereas no significant effects were observed for free Ac2‐26. These results showed that AON promoted epithelial barrier recovery.

#### 2.8.7 AON Expedited the Resolution of Inflammation

Using a peritonitis model, we examined the proresolving capacity of AON.^43^ Immediately after challenging with zymosan by intraperitoneal (i.p.) injection, mice were treated with different Ac2‐26 formulations and then the peritoneal exudates were collected after 6 h exposure to zymosan. AON prevented zymosan‐induced neutrophil infiltration by 30%, while treatment with free Ac2‐26 and APN only showed a reduction of ≈4% and ≈18%, respectively (Figure 9D,E). We further evaluated the effect of AON on the resolution of inflammation. After i.p. injection of zymosan, neutrophil infiltration maximized at 6 h (*Ψ*_max_ = ≈9.0 × 10^6^) (Figure 9F), and then declined to a value of ≈4.0 × 10^6^ at 24 h. The calculated resolution interval (*R*_i_) was ≈15 h (Figure 9G). Local AON treatment significantly reduced the number of infiltrating neutrophils at each time point examined after zymosan injection, with a *Ψ*_max_ of ≈6.3 × 10^6^ at 6 h and a *R*_i_ of 11 h (Figure 9H).

Treatment with AON also significantly decreased the levels of TNF‐α and IL‐1β, indicating that inflammatory responses were mitigated, while APN had no significant anti‐inflammatory effects in vivo (Figure S30A,B, Supporting Information). While free Ac2‐26 and APN partially reduced the expression of MPO, AON treatment showed a significantly better suppression (Figure S30C, Supporting Information). Furthermore, treatment with different formulations remarkably inhibited the generation of MDA and H_2_O_2_ (Figure S30D,E, Supporting Information), with the most effective suppression by AON.

#### 2.8.8 Regulation of the Gut Microbiota by AON

**Figure 10:**
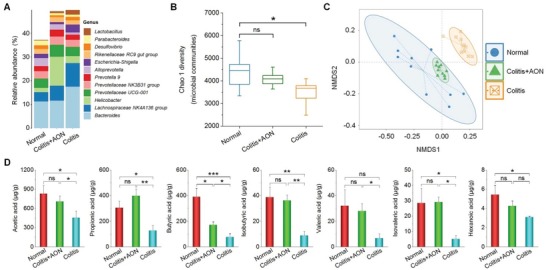
Analysis of the gut microbiota and quantification of microbiota‐derived short‐chain fatty acids (SCFAs) in colitis mice after treatment with AON. A) The changes of genus level in fecal microbiota composition. B) Box‐and‐whisker plot of Chao 1 alpha diversity of the microbial community in the feces based on 16S profiling. Boxes show median, first, and third quantiles, and whiskers denote the minimum to maximum range. C) Nonmetric multidimensional scaling (NMDS) analysis of the microbiota composition. D) The concentrations of fecal SCFAs. Data are presented as mean ± SE (*n* = 10). *p < 0.05, **p < 0.01, ***p < 0.001; ns, no significance.

Using 16S rRNA gene sequencing, we investigated the composition of gut microbiota in mice. Compared to the normal mice, mice with DSS‐induced colitis showed a markedly increased proportion of *Escherichia–Shigella* (Figure S31, Supporting Information and **Figure** **10**A), which are well‐known infective pathogens that contribute to the development of colitis.^44^ In contrast, we found a significant decrease of the Prevotellaceae family, which use nondigestible carbohydrates in the colon to produce short‐chain fatty acids (SCFAs) to serve as energy substrates and exert anti‐inflammatory effects.^45^ AON treatment reduced the expansion of *Escherichia–Shigella* and increased Prevotellaceae levels. Analysis of Chao1 alpha diversity, an indicator of species richness, showed that colitis mice were characterized by a significant drop in taxa richness, suggesting considerably decreased community diversity and structure. This is consistent with a previous finding on patients with active IBD.^46^ In contrast, no differences were observed in biodiversity between the untreated normal control and the AON treatment group (Figure 10B). Nonmetric multidimensional scaling (NMDS) analysis of unweighted UniFrac distance was used to compare the degree of similarity of gut microbial composition in three groups. The result showed a clear difference in the fecal bacterial phylogenetic architecture between the DSS‐treated and normal groups (Figure 10C). Moreover, components of the fecal microbiota of the AON‐treated mice were similar to those of normal mice. These results suggested that AON treatment can shift the microbial community profile from a dysbiotic state toward homeostasis for mice with DSS‐induced colitis. According to in vitro and in vivo biological studies as aforementioned, AON remodeled the gut microbiota largely by normalizing oxidative and proinflammatory microenvironment.

Among gut microbiota‐generated metabolites, SCFAs, which are solely metabolized from indigestible carbohydrates by the intestinal microbiota, have been demonstrated to be intimately associated with a reduced risk of IBD.^45^ SCFAs can activate G protein‐coupled receptors and also inhibit histone deacetylase activity,^47^, ^48^ thereby down‐regulating proinflammatory cytokine production by macrophages. We further determined the contents of seven SCFAs in the feces of mice subjected to different treatments. In the colitis group, SCFAs significantly decreased compared to those of the normal group (Figure 10D). Such a decrease was reversed by treating with AON, in line with the increase of Prevotellaceae (Figure 10A). Taken together, these data suggested that AON may exert its anti‐inflammatory effects and improve bowel health by shaping the gut microbiota and increasing SCFAs production.

### 2.9 Safety Profile of Orally Administered AON

We first evaluated the cytotoxicity of different NPs using RAW264.7 macrophages. Relatively high cell viability was observed for ON, AON, and APN across a broad range of concentrations (Figure S32, Supporting Information). Our results are in line with the fact that APN is derived from an FDA‐approved material PLGA.

Then potential side effects after oral administration of a high dose of AON (5.0 g kg^−1^) were examined in vivo. At the predefined time points, no significant weight differences were found between control and AON‐treated groups (Figure S33A, Supporting Information). Similarly, no changes were found between two groups on the organ indexes of major organs including heart, liver, spleen, lung, and kidney (Figure S33B, Supporting Information). Representative hematological parameters were in normal ranges for mice treated with AON (Figure S33C–E, Supporting Information). Quantification of biomarkers relevant to hepatic and kidney functions revealed no abnormal variations in the AON treated group compared to the control group (Figure S33F–I, Supporting Information). Consistent with these results, further examination on H&E sections of the gastrointestinal tissues and major organs, which are closely related to absorption and excretion of orally administered AON, showed no discernable injuries in these organs (Figure S34, Supporting Information). All these results suggested that AON displayed good safety profile for oral administration.

## 3 Discussion

Gut homeostasis is maintained by complex interactions between the mucosal immune cells, intestinal epithelium, and gut microbiota.^49^ Therefore, new therapies that can remodel proinflammatory microenvironment and reverse gut dysbiosis are promising for the IBD treatment. As an active N‐terminal peptide of an endogenous proresolving protein annexin A1, Ac2‐26 is promising for the management of IBD, due to its multiple anti‐inflammatory effects. However, it remains unclear whether its effectiveness can be retained after oral delivery that is the most preferred strategy for therapy of the localized inflammatory disorders in the gastrointestinal tract,^25^, ^26^, ^27^, ^28^, ^50^ due to its diverse advantages, such as convenience, desirable cost‐effectiveness, excellent safety profile, and high patient compliance.

Unfortunately, orally delivered Ac2‐26 was inactive, as demonstrated by our results in mice with DSS‐induced colitis. This was mainly resulted from hydrolysis of Ac2‐26 in the harsh environment of the gastrointestinal tract, which is a major challenge for oral delivery of peptides/proteins.^51^ Consistently, we found complete hydrolysis of Ac2‐26 after it was incubated in different solutions simulating gastric/intestinal fluids. Our previous studies substantiated that NPs based on a ROS‐responsive material OxbCD can be selectively hydrolyzed under inflammatory conditions with overexpressed ROS, while they are stable in the stomach.^30^, ^31^ Compared to ROS‐responsive nanocarriers based on other materials, such as poly(propylene sulfide), selenium‐containing copolymers, polydopamine, and materials bearing polythioether ketal or thioketal linkers,^52^, ^53^ OxbCD‐derived NPs are highly sensitive to H_2_O_2_.^30^, ^32^ More importantly, our comprehensive in vitro and in vivo studies have revealed good safety profile for nanocarriers based on OxbCD.^30^, ^31^, ^32^, ^33^, ^34^, ^36^, ^54^, ^55^ Consequently, we hypothesize that the ROS‐responsive nanocarrier ON can revive orally delivered Ac2‐26 by protecting it from degradation and selective release in the inflamed colon of IBD. Using a modified nanoprecipitation technique, Ac2‐26 was efficiently loaded into ON, resulting in a ROS‐triggerable nanotherapy AON with a spherical shape and tailored size. At pH 7.4 and 1.0 × 10^−3^ m H_2_O_2_, AON rapidly hydrolyzed, concomitant with efficient Ac2‐26 release. In addition, Ac2‐26 in AON was stable against hydrolysis in different simulated gastric/intestinal fluids.

After oral administration, we found significant localization and accumulation of AON in the inflamed colon of mice with DSS‐induced colitis. The enhanced accumulation of AON in colon tissues should be mainly due to the reduced thickness of the mucus layer and the destruction of the intestinal barrier under inflammatory conditions,^56^, ^57^ agreeing with previous studies on other NPs.^22^, ^27^, ^28^, ^58^, ^59^ Moreover, flow cytometry analysis revealed AON internalization by macrophages and neutrophils in the colonic mucosal tissue. Consistently, AON treatment of mice with acute or chronic colitis afforded desirable outcomes, as manifested by significantly decreased weight loss, reduced DAI, and well maintained colon length as well as notably improved colonic morphology and microstructure. Compared to free Ac2‐26, ON, Ac2‐26/ON mixture, and a nonresponsive control nanotherapy APN, AON significantly alleviated the symptoms of colitis.

We further examined the mechanisms underlying the therapeutic effects of AON in vitro and in vivo. Both Ac2‐26 and its nanotherapies effectively inhibited macrophage apoptosis as well as reduced ROS generation and inflammatory response in stimulated macrophages. On the other hand, high and sustained neutrophil influx may lead to chronic inflammatory diseases.^60^ Thus, inhibition of neutrophil trafficking to the inflamed tissue is an essential process for the resolution of inflammation.^61^ We found that AON can reduce neutrophil transmigration in vitro, which might be attributed to the ability of Ac2‐26 to cause transient calcium fluxes and l‐selectin shedding in neutrophils as well as inhibit the adhesiveness of β1 and β2 integrins by downregulating their affinity and valency.^62^, ^63^

In addition, macrophage phagocytic clearance of apoptotic neutrophils plays an important role in resolving inflammation, since this process can prevent excessive neutrophil activation and the exposure of tissues to noxious intracellular contents of neutrophils or neutrophil extracellular traps.^64^ AON enhanced the clearance of apoptotic neutrophils by macrophages. This effect should be associated with Ac2‐26‐mediated opsonization of apoptotic cells, probably by interacting with phosphatidylserine on the surface of apoptotic cells, acting as bridging molecules, linking apoptotic cells to phagocytes, and promoting phagocytosis.^60^ We found that AON exerted its anti‐inflammatory and proresolving actions through FPR2, and the antiapoptotic activity in macrophages was largely attributed to FPR1 activation. Moreover, AON effectively switched macrophages from a proinflammatory M1 phenotype toward an anti‐inflammatory M2 phenotype, which further reduced production of proinflammatory cytokines and increased release of proresolving molecules.^40^ Both anti‐inflammatory and antimigratory properties of AON were validated by in vivo studies in mice with acute and chronic colitis or zymosan‐induced peritonitis. Also, oral administration of AON efficiently restored epithelial barrier function that is very important for recovery of colitis, thereby preventing subsequent neutrophil influx and inhibiting exacerbation of mucosal inflammation.^65^

In addition to the above mentioned proinflammatory molecules/cells, increasing evidence has demonstrated that the gut microbiota is pivotal for shaping the gut immune system, thereby contributing to the maintenance of gut homeostasis.^66^ Dysbiosis of the gut microbiota is intimately related to IBD susceptibility.^67^ Our study revealed a high abundance of potentially infective *Escherichia–Shigella* and low abundance of Prevotellaceae in DSS‐induced mice, which should partly contribute to the development of colitis. Therapy with AON shifted the microbial community profile from a dysbiotic state toward a normal state with improved bacterial diversity, which is in accordance with the increased levels of SCFAs after treatment. Commensal bacteria‐derived SCFAs can induce the differentiation of colonic regulatory T cells,^68^ and they are also important energy sources for intestinal epithelial cells to produce mucin and antimicrobial peptides.^69^ Our data suggested that both gut microbiome remodeling and improved SCFAs collectively contributed to the therapeutic role of AON in vivo.

## 4 Conclusion

In summary, we engineered a proresolving peptide Ac2‐26 containing, ROS‐responsive nanotherapy AON, to effectively target inflamed colons and treat IBD in mice with acute and chronic colitis, by regulating the inflammatory microenvironment and microbial community. Our AON has showed excellent biosafety in a mouse model in vivo and is promising for the development of site‐specific therapy for IBD and other inflammatory diseases.

## Conflict of Interest

The authors declare no conflict of interest.

## Supporting information

Supplementary
