# Supplementary material for: A Proresolving Peptide Nanotherapy for Site‐Specific Treatment of Inflammatory Bowel Disease by Regulating Proinflammatory Microenvironment and Gut Microbiota
Source: Adv Sci (Weinh). 2019 Aug 1;6(18):1900610. doi: 10.1002/advs.201900610 (PMC6755521; doi:10.1002/advs.201900610)
Supplement: Supplementary file 1 — Supplementary [file ADVS-6-1900610-s001.pdf]

Copyright WILEY-VCH Verlag GmbH & Co. KGaA, 69469 Weinheim, Germany, 2019.

ADVANCED SCIENCE
Open Access

# Supporting Information

for *Adv. Sci.,* DOI: 10.1002/advs.201900610

# A Proresolving Peptide Nanotherapy for Site-Specific Treatment of Inflammatory Bowel Disease by Regulating Proinflammatory Microenvironment and Gut Microbiota

*Chenwen Li, Yang Zhao, Juan Cheng, Jiawei Guo, Qixiong Zhang, Xiangjun Zhang, Jiong Ren, Fengchao Wang, Jun Huang, Houyuan Hu, Ruibing Wang,* and Jianxiang Zhang**

Copyright WILEY-VCH Verlag GmbH & Co. KGaA, 69469 Weinheim, Germany, 2019.

# Supporting Information

**A Proresolving Peptide Nanotherapy for Site-Specific Treatment of Inflammatory Bowel Disease by Regulating Proinflammatory Microenvironment and Gut Microbiota**

Chenwen Li, Yang Zhao, Juan Cheng, Jiawei Guo, Qixiong Zhang, Xiangjun Zhang, Jiong Ren, Fengchao Wang, Jun Huang, Houyuan Hu, Ruibing Wang*, and Jianxiang Zhang*

C. W. Li, Y. Zhao, J. Cheng, Dr. J. W. Guo, Dr. Q. X. Zhang, Prof. J. X. Zhang
Department of Pharmaceutics, College of Pharmacy, Third Military Medical University, Chongqing 400038, China
E-mail: jxzhang1980@gmail.com; jxzhang@tmmu.edu.cn

J. Ren, Prof. F. C. Wang
State Key Laboratory of Trauma, Burns and Combined Injury, Institute of Combined Injury, College of Preventive Medicine, Third Military Medical University, Chongqing 400038, China

X. J. Zhang, Prof. R. B. Wang
State Key Laboratory of Quality Research in Chinese Medicine, Institute of Chinese Medical Sciences, University of Macau, Taipa, Macau, China
E-mail: rwang@umac.mo

Prof. J. Huang
Institute for Molecular Engineering, University of Chicago, Chicago, IL 60637, USA

Prof. H. Y. Hu
Department of Cardiology, Southwest Hospital, Third Military Medical University, Chongqing 400038, China

**Supplementary Materials and Methods**

**Materials.** β-Cyclodextrin (β-CD) and lecithin (from soybean) were purchased from Tokyo Chemical Industry Co., Ltd. (Tokyo, Japan). Ac2-26 (Ac-AMVSEFLKQAWFIENEEQEYVQTVK), FITC-labeled Ac2-26 (FITC-Ac2-26), Cy5-labeled Ac2-26 (Cy5-Ac2-26), and Cy7.5-labeled Ac2-26 (Cy7.5-Ac2-26) were synthesized by ChinaPeptides Biological Technology Co., Ltd (Shanghai, China). 4-(Hydroxymethyl)phenylboronic acid pinacol ester (PBAP), 4-dimethylaminopyridine (DMAP), and 1,1'-carbonyldiimidazole (CDI) were purchased from Acro Organics (U.S.A.). Poly(lactide-co-glycolide) (PLGA, 50:50) with an intrinsic viscosity of 0.50-0.65 was purchased from Polysciences, Inc. (U.S.A.). 1,2-Distearoyl-sn-glycero-3-phosphoethanolamine-N-[methoxy(polyethylene glycol)-2000] (DSPE-PEG) was purchased from Corden Pharma (Switzerland). Penicillin, streptomycin, fetal bovine serum (FBS), and Dulbecco's modified eagle medium (DMEM) were provided by Gibco (Waltham, U.S.A.). 4,6-Diamidino-2-phenylindole (DAPI) and LysoTracker Red were supplied by Invitrogen (U.S.A.). Dextran sulfate sodium (DSS, 35000 Da) was supplied by MP Biomedical (U.S.A.). FITC-Dextran (average $M_w$ 4,000 kDa), phorbol 12-myristate 13-acetate (PMA), 2′,7′-dichlorofluorescin diacetate (DCFH-DA), lipopolysaccharide (LPS) from Escherichia coli were purchased from Sigma-Aldrich (U.S.A.). APC Annexin V Apoptosis Detection Kit with PI and BV510-conjugated rat anti-mouse F4/80 antibody were purchased from Biolegend (U.S.A.). The V450-conjugated rat anti-mouse CD11b antibody, PE-conjugated rat anti-mouse Ly6G, FITC-conjugated rat anti-mouse F4/80 antibody, PE-conjugated rat anti-mouse CD86 antibody, and APC-conjugated rat anti-mouse CD206 antibody were obtained from BD Biosciences (U.S.A.). 3,3′-Dioctadecyloxacarbocyanine perchlorate (DiO) was purchased from Beyotime Institute of Biotechnology (China). All ELISA kits were obtained from Boster Biological Technology Co., Ltd (Wuhan, China). Oxidative stress-related kits and BCA protein assay kit were obtained from Nanjing Jiancheng Bioengineering Institute (Nanjing, China). All the other reagents are commercially available and used as received.

**Synthesis and characterization of a ROS-responsive material based on β-CD.** A ROS-responsive material (OxbCD) was synthesized according to our previously reported methods (Figure S3A).[1] Specifically, PBAP (1.11g, 4.72 mmol) was dissolved in anhydrous dichloromethane (DCM) (10 mL), into which 1.53 g CDI (9.44 mmol) was added. After 1 h of reaction at room temperature, 9 mL of DCM was added into the mixture, followed by washing with 10 mL of deionized water three times. The organic phase was further washed with saturated NaCl solution, dried over $Na_2SO_4$, and concentrated in vacuum to obtain CDI-activated PBAP. Subsequently, 1.52 g activated PBAP and 250 mg β-CD were co-dissolved in 20 mL of anhydrous DMSO. After DMAP (0.80 g, 6.55 mmol) was added, the obtained mixture was magnetically stirred at room temperature overnight. The final product was obtained by precipitation from water (80 mL), and collected by centrifugation. After thorough rinsing with deionized water, the sample was lyophilized to give a white powder. The obtained OxbCD was characterized by $^1$H NMR and Fourier transform infrared (FT-IR) spectroscopy.

**Fabrication of nanoparticles by a nanoprecipitation/self-assembly method.** A modified nanoprecipitation/self-assembly method was employed to prepare Ac2-26-loaded OxbCD nanoparticles (Ac2-26/OxbCD NP, defined as AON).[1] Briefly, 50 mg OxbCD was dissolved in 2 mL of methanol, into which 100 μg Ac2-26 dissolved in DMSO was added. The obtained solution was added dropwise into 15 mL of deionized water containing 9 mg DSPE-PEG and 6 mg lecithin that was preheated at 65°C. After 2 h of incubation, solidified Ac2-26-loaded NPs were harvested by centrifugation at 21752g and rinsing with deionized water three times. Following similar procedures, OxbCD NP (ON), FITC-Ac2-26-containing OxbCD NP (FITC-AON), Cy7.5-Ac2-26-containing OxbCD NP (Cy7.5-AON), Cy5-Ac2-26-containing OxbCD NP (Cy5-AON), and Ac2-26-containing PLGA NP (APN) were fabricated.

**Characterization of nanoparticles.** Particle size and zeta-potential measurements were conducted on a Malvern Zetasizer Nano ZS instrument at 25°C. Transmission electron microscopy (TEM) observation was carried out on a TECNAI-10 microscope (Philips, Netherlands), operating at an acceleration voltage of 80 kV. Scanning electron microscopy (SEM) was conducted on a FIB-SEM microscope (Crossbeam 340, Zeiss).

The loading content of FITC-Ac2-26 in different NPs was determined by fluorescence measurement of FITC-Ac2-26. To this end, the sample of lyophilized FITC-Ac2-26-loaded NPs was dispersed in methanol for extraction of FITC-Ac2-26. Then the concentration of FITC-Ac2-26 was quantified by fluorescence spectroscopy (F-7000, Hitachi, Japan). The excitation wavelength was set at 494 nm, while the emission wavelength was 518 nm. The concentration of FITC-Ac2-26 was calculated according to a standard curve established with a series of FITC-Ac2-26 aqueous solutions with predetermined concentrations.

To quantify the content of Ac2-26 in different NPs, lyophilized Ac2-26-loaded NPs were dispersed in methanol to extract Ac2-26. Then high-performance liquid chromatography (HPLC, LC-20A, Shimadzu) was performed to determine the concentration of Ac2-26. The detection wavelength was 220 nm, while the mobile phase consisted of aqueous solution containing 0.1% trifluoroacetic acid (TFA) and acetonitrile containing 0.1% TFA (from 40% to 70% in 8 min), with a flow rate of 1.0 mL/min.

**In vitro hydrolysis and release study.** In vitro hydrolysis of Ac2-26-containing NPs derived from OxbCD or PLGA was performed at 37°C in PBS buffers (0.01 M, pH 7.4) with or without 1.0 mM of hydrogen peroxide. The degree of hydrolysis was calculated based on the transmittance values. At various time points, the transmittance was measured at 500 nm.

For in vitro release tests, 4.0 mL of aqueous solution containing 10.0 mg freshly fabricated FITC-Ac2-26-containing NPs was placed into dialysis tubing (MWCO: 8000-14000 Da), which was immersed in 40 mL of PBS (0.01 M, pH 7.4) with or without 1.0 mM of hydrogen peroxide. At predetermined time intervals, 2 mL of release medium was withdrawn, and the same volume of fresh medium was supplemented. The concentration of Ac2-26 in the release medium was quantified by fluorescence spectroscopy.

Through similar procedures, drug release in fluids simulating the alimentary tract environment was also examined. For this purpose, different NPs were first incubated in simulated gastric fluid at pH 1.2 for 2 h. Then, the dissolution medium was changed to PBS (0.01 M, pH 7.4) containing 1.0 mM of hydrogen peroxide.

**Stability of Ac2-26 in physiological fluids.** Simulated gastric fluid was prepared by dissolving pepsin (pepsin A from porcine gastric mucosa) in saline to a final concentration of 0.032 mg/mL, which was adjusted to pH 1.2 with concentrated HCl. In addition, simulated intestinal fluid was freshly prepared by dissolving trypsin (from porcine pancreas) in 0.02 M PBS to a final concentration of 0.1 mg/mL, followed by adjustment to pH 7.4 with 0.2 mol/L NaOH.

On the other hand, to obtain murine gastric and intestinal fluids, C57BL/6J mice were euthanized. Then the whole stomach and colon were separately washed with 5 mL of ice-cold PBS. After centrifugation, the supernatant was collected. To obtain stomachic and colonic homogenates, the stomach and colon from sacrificed mice were homogenized in 3 mL of ice-cold PBS after washing with PBS. The homogenate was centrifuged and the supernatant was collected.

Subsequently, 100 μL of aqueous solution containing AON or free Ac2-26 was incubated at 37°C for 2 h in 0.2 mL of various fluids including simulated gastric fluid, simulated intestinal fluid, murine gastric fluid, and colonic fluid, as well as stomachic and colonic homogenates. Then Ac2-26 in AON was extracted by methanol. The content of Ac2-26 was quantified by HPLC as aforementioned.

**Animals.** All animal experiments were performed in line with the Guide for the Care and Use of Laboratory Animals proposed by National Institutes of Health. All procedures and protocols were approved by the Animal Ethics Committee at Third Military Medical University (Chongqing, China). Female C57BL/6J mice (18-20 g) and male BALB/c mice (18-20 g) were obtained from the Animal Center of the Third Military Medical University. Male B6.129P2-*Il*10$^{tm1Cgn}$/*J* (IL-10$^{-/-}$) mice were obtained from the Jackson Laboratory. Animals were housed in standard mouse cages under standard conditions, with ad libitum access to water and food. All mice were acclimatized for at least one week before further experiments.

**Study on biodistribution of Ac2-26 NPs in mice with acute colitis by ex vivo imaging.** Acute ulcerative colitis in mice was induced by addition of 3% (w/v) DSS to the drinking water for 7 days.[2] Cy7.5-labeled Ac2-26 (Cy7.5-Ac2-26) was used to evaluate the localization and biodistribution of Ac2-26-loaded NPs after oral administration. After 7 days of treatment with DSS, mice received a single oral administration of Cy7.5-Ac2-26-loaded NPs (containing 0.04 mg of Cy7.5). At each defined time point, mice were euthanized. The colon segments and major organs including liver, spleen, kidney, intestine, and mesenteric lymph node (MLN) were isolated. Blood was also collected for analysis. Ex vivo imaging was carried out with a living imaging system (IVIS Spectrum, PerkinElmer, U.S.A.). The fluorescence intensity was analyzed by the Living Imaging software.

**Study on biodistribution of Ac2-26 NPs in mice with acute colitis by HPLC.** After 7 days of treatment with 3% DSS in the drinking water, mice received a single oral administration of Ac2-26 or AON (containing 50 μg of Ac2-26). At predetermined time points, mice were euthanized. Whole blood, the colon tissues, and major organs were collected. The plasma was obtained by centrifugation at 2000*g* for 10 min. For different tissues, they were homogenized in PBS and centrifuged, and the supernatant was collected. After the proteins were precipitated via methanol, the concentration of Ac2-26 was quantified by HPLC.

**Observation of the localization of Ac2-26 NPs in the inflamed colon.** The localization of orally delivered NPs in the colon of mice with DSS-induced colitis was also observed by CLSM to evaluate the selective accumulation of AON in the inflamed colon. Briefly, acute colitis was induced in mice as aforementioned. After 7 days, mice received orally administered FITC-AON or free FITC-Ac2-26 (the dose of FITC-Ac2-26 was 4.0 mg/kg). Mice were euthanized after 4 h. Colons were removed and embedded in Tissue-Tek O.C.T. Compound and frozen at -80°C for subsequent experiments. After 7 μm-thick colon cryosections were stained with DAPI, images were captured using a Zeiss confocal microscope.

**Cellular uptake of Ac2-26 NPs in the inflamed colonic tissues of colitis mice.** To further investigate cellular uptake of Ac2-26 NPs, the experiments were performed in both healthy and colitis mice. Ulcerative colitis in mice was induced as mentioned above. Mice were orally administered with Cy5-Ac2-26-loaded NPs at 0.24 mg/kg of Cy5-Ac2-26. After 6 h, mice were euthanized. The colon was resected and suspended into 5 mL of HBSS containing 5% FBS, type IV collagenase (1 mg/mL), and EDTA (2 μmoL/mL), followed by shaking at 220 rpm for 20 min at 37°C. This process was repeated three times. Single-cell suspensions were obtained by passing suspensions through 70 μm cell strainers. The cells were stained with V450-conjugated rat anti-mouse CD11b antibody, PE-conjugated rat anti-mouse Ly6G antibody, BV510-conjugated rat anti-mouse F4/80 antibody, or isotype controls for 20 min. Flow cytometric analysis was performed on a BD FACSVerse flow cytometer. Data were analyzed using the FlowJo v10 software.

**Treatment of acute colitis in mice.** Acute ulcerative colitis in mice was induced by drinking water containing DSS as aforementioned. First, we examined therapeutic effects of free Ac2-26 after oral administration. In this case, colitis mice were randomly assigned to two groups: a saline-treated group and free Ac2-26-treated group (at 250 μg/kg). Both saline and Ac2-26 were orally administered daily during 7 days of DSS treatment. In addition, healthy mice were used as the normal control. Mice were observed daily, and changes in the body weight, visible stool consistency, and fecal bleeding were evaluated. Disease activity index (DAI) is defined as the summation of the stool consistency index (0-3), fecal bleeding index (0-3), and weight loss index (0-4).[3]

In another cohort study, animals were randomized to different experimental groups. Healthy mice in the Normal group were not treated, while colitis mice in the Colitis, ON, APN, AON groups were orally administered with saline, blank OxbCD NP, APN, and AON, respectively. For all diseased mice, daily administration was performed during 7 days of DSS treatment. The dose was 250 μg/kg of Ac2-26 for all Ac2-26-containing formations. Then similar evaluations as mentioned above were followed to examine efficacies.

**Induction of chronic colitis and drug treatments in mice.** To induce chronic colitis in mice, animals were administered with 1.5% (w/v) DSS in their drinking water ad libitum for three consecutive 5-day periods: days 1-5, 11-15, and 21-25.[4] After each period of DSS administration, the animals were treated with saline, Ac2-26, APN, or AON by oral gavage. The dose was 250 μg/kg of Ac2-26 for all Ac2-26-containing formations. The healthy mice in the control group were allowed to drink water. The body weight and DAI of each animal were monitored daily.

In a separate study, mice were randomized to different experimental groups. Healthy mice in the normal group were not treated, while mice with chronic colitis induced as mentioned above were orally administered with saline, ON, Ac2-26, Ac2-26/ON mixture, and AON, respectively. The dose was 250 μg/kg of Ac2-26 for all Ac2-26-containing formations. Then similar procedures were followed.

For the treatment of IL-$10^{-/-}$ mice with spontaneous chronic colitis, IL-$10^{-/-}$ mice were randomized to three groups, which were orally treated with saline, APN, or AON. The dose was 250 μg/kg of Ac2-26 for all Ac2-26-containing formations. In addition, healthy C57BL/6J mice were administered with saline in the normal group. The treatment protocols are similar to those used in mice with DSS-induced chornic colitis.

**Evaluations of therapeutic effects of Ac2-26 NPs for the treatment of colitis.** After various treatments, mice were euthanized, and the entire colon (from the cecum to the rectum) was collected. The colon length was measured and gently washed with saline. Then, 1 cm of the distal section was used for histological assessment. The remaining section was taken for measuring the levels of tumor necrosis factor-α (TNF-α), interferon-γ (IFN-γ), interleukin-1β (IL-1β), malondialdehyde (MDA), and hydrogen peroxide as well as myeloperoxidase (MPO) activity. Blood samples were collected for quantification of hematological parameters (Sysmex KX-21, Sysmex Co., Japan) and biochemical markers relevant to liver/kidney functions (Roche Cobas C501, Roche Co., Switzerland). Major organs including heart, liver, spleen, lung, kidney, stomach, intestine, and MLN were harvested and weighed.

Single cell suspensions of the colonic tissues were prepared as aforementioned. The cells were stained with V450-conjugated rat anti-mouse CD11b antibody, PE-conjugated rat anti-mouse Ly6G antibody, BV510-conjugated rat anti-mouse F4/80 antibody, or isotype controls for 20 min. The number of neutrophils and macrophages were assessed by flow cytometry using a BD FACSVerse flow cytometer.

**Mini-endoscopic imaging.** For direct visualization of DSS-induced colonic mucosal damage, an endoscopic video system for mice was used. The experimental endoscope setup consisted of a

miniature endoscope (a scope with outer diameter of 1.9 mm), a xenon light source, a triple chip camera (Karl Storz, Germany), and an air supply to achieve regulated inflation of the mouse colon. Before experimentation, mice were anesthetized by inhalation of isoflurane (RWD Life Science, China). The endoscopic procedure was viewed on a color monitor and digitally recorded on a tape.

**Quantification of the MDA and hydrogen peroxide levels.** Both hydrogen peroxide and MDA in different colon tissues were determined according to the manufacturer's instructions. Briefly, stored tissues were homogenized in phosphate buffer (pH 7.4) at a ratio of 1:10 (per mg in 10 mL). The supernatant was collected by centrifugation at 10,621g for 15 min at 4°C. MDA was detected using the thiobarbituric acid-reacting substance assay as previously described.[5] In addition, the total protein concentration was measured by the BCA method. The results are expressed as nmol per mg protein. For hydrogen peroxide, its concentration was quantified following the standard protocols provided by the manufacturer.

**Quantification of inflammatory mediators in the colonic tissue.** The colonic tissues were collected immediately after mice were euthanized and homogenized in cold PBS. After centrifugation at 10,621g for 15 min at 4°C, the levels of TNF-α, IL-1β, IFN-γ, and MPO in the supernatant were measured by ELISA analysis.

**Histological assessment.** In brief, 1 cm of the distal colon was fixed in 4% (v/v) buffered formalin and embedded in paraffin. Tissue sections with a thickness of 7 μm were stained with hematoxylin and eosin (H&E). Histology of the colon was evaluated by optical microscopy.

**Immunofluorescence analysis.** The sections of colon tissues were blocked with 1% bovine serum albumin for 30 min at room temperature, and then incubated with antibodies to cytokeratin 18 (CK18) at 37°C for 30 min. After 24 h, the slices were incubated with the secondary antibody of Cy3-conjugated goat anti-rat IgG for another 30 min. After the nuclei were stained with DAPI, the sections were imaged by confocal microscopy.

**Survival rate of colitis mice after different treatments.** The survival rate of mice was determined by replacing drinking water with 3% (w/v) DSS for 12 days. Different formulations were orally administered daily on day 1 until day 12. The number of surviving mice was counted until day 12.

**Cell culture.** RAW264.7 murine macrophage cell line was purchased from the Institute of Biochemistry and Cell Biology (Shanghai, China). Cells were cultured in DMEM supplemented with 10% FBS with penicillin/streptomycin at 37°C in a humidified atmosphere containing 5% $CO_2$.

**Cytotoxicity of various nanoparticles.** To evaluate cytotoxicity of different NPs, RAW264.7 cells were seeded at a density of $1\times10^4$ cells per well in 96-well plates. After 24 h, the culture medium was replaced by 100 μL of fresh medium containing gradient concentrations of blank and Ac2-26-containing NPs and incubated for 24 h. The cell viability was quantified by MTT assay.

**Studies of intracellular ROS generation in macrophages.** To analyze the inhibitory effect of AON on intracellular ROS generation in PMA-stimulated macrophages, RAW264.7 cells were seeded in a 12-well plate ($2 \times 10^5$ cells per well) and incubated overnight. After the cells were pretreated with medium containing various NPs or free Ac2-26 (1 μM) for 4 h at 37°C, the medium was removed and replaced with PMA (100 ng/mL), followed by 1 h of incubation. The cells were then washed with PBS and incubated with 10 μM DCFH-DA (a ROS-sensitive fluorescent dye) for 30 min. Fluorescence intensity was measured via FACS.

To directly observe ROS-induced DCF fluorescence, RAW264.7 cells were cultured in glass-bottom cell culture dishes at a density of $5 \times 10^4$ cells/plate for 24 h before treatment. Through similar procedures as mentioned above, fluorescent images were acquired by CLSM (Leica, Germany).

**Apoptosis assay by flow cytometry.** Apoptosis analysis was conducted using Annexin V-APC (Annexin V) and propidium iodide (PI) detection kit according to the manufacture's protocol. Briefly, RAW264.7 cells were seeded in a 12-well plate at a density of $2 \times 10^5$ cells/well and incubated overnight. The medium was then replaced with fresh medium containing various formulations and incubated for 4 h. Cells were treated with 200 μM $H_2O_2$ for 12 h. Subsequently, cells were stained with annexin V-APC conjugate and PI, and the fluorescence intensity was measured by flow cytometry. Annexin $V^-$/$PI^-$, Annexin $V^+$/$PI^-$, and Annexin $V^+$/$PI^+$ represented live cells, apoptotic cells, and necrotic cells, respectively.

**In vitro anti-inflammation tests.** RAW264.7 macrophages were seeded in 12-well plates at a density of $2 \times 10^5$ cells/well and incubated overnight. After co-culture with various NPs for 4 h, cells were stimulated with LPS (100 ng/mL) for another 6 h. The supernatant was collected to determine the TNF-α concentration by ELISA kit.

**In vitro anti-migration activity of Ac2-26-containing NPs in neutrophils.** To induce production of neutrophils, 1.0 mL of aqueous solution containing thioglycollate (3.0 wt%) was intraperitoneally (i.p.) administrated to BALB/c mice.[6] After 4 h, mice were euthanized and peritoneal exudates containing neutrophils (> 90%) were collected by lavaging with 5 mL of sterile Hank's balanced salt solution (HBSS). The suspension was centrifuged at 400g for 10 min.

A transwell assay was performed to investigate anti-migration activity of Ac2-26 NPs. Briefly, isolated neutrophils ($2 \times 10^5$ cells) were incubated with various NPs or free Ac2-26 (1.0 μM) on the upper chamber of the transwell system. The lower chamber was filled with a FBS-free culture medium. After 1 h of incubation at 37°C, the cells in the lower chamber were harvested and counted by optical microscopy. At least five fields were observed.

**Phagocytosis assay.** Neutrophils were isolated as aforementioned, fluorescently labeled using DiO membrane dye following manufacturer's instructions, and aged in DMEM with 10% FBS for 24 h in order to undergo apoptosis as previously described.[7] RAW264.7 macrophages were seeded in 12-well plates at a density of $2 \times 10^5$ cells/well and incubated overnight. The medium was then replaced with fresh medium containing various formulations and incubated for 1 h. Cells were co-incubated with DiO-labeled apoptotic neutrophils ($1 \times 10^6$ neutrophils/well) at 37°C for another 1 h. Subsequently, non-ingested cells were removed by three times of washing with cold PBS. The macrophage phagocytosis capability was assessed by quantifying fluorescence via flow cytometry.

**Macrophage polarization.** RAW264.7 macrophages were stimulated in medium containing 200 ng/mL LPS with 2.5 ng/mL IFN-γ for 24 h. Then cells were treated with different formulations for 8 h. Subsequently, macrophages were labeled with different combinations of antibodies to identify M1 and M2 macrophages. FITC-conjugated rat anti-mouse F4/80 antibody and PE-conjugated rat anti-mouse CD86 antibody were used for staining M1 macrophages, while M2-like macrophages were labeled with FITC-conjugated rat anti-mouse F4/80 antibody and APC-conjugated rat anti-mouse CD206 antibody. The corresponding isotype controls were also used. After washing twice with PBS, samples were analyzed by flow cytometry.

**Epithelial wound healing assay.** Acute colitis was induced in mice as aforementioned, and then randomly assigned mice received different formulations by daily oral administration. After 7 days of

treatment, each mouse received FITC-dextran by oral gavage at 0.6 mg/g. After 4 h, blood was sampled and centrifuged at 10,000g for 10 min at 4°C. The supernatant was diluted with PBS (1:3, v/v), and the FITC fluorescence was measured, after excitation at 485 nm and emission 535 nm. Standard curves were obtained by diluting FITC-dextran in non-treated plasma with PBS (1:3, v/v).

**Fecal DNA extraction and 16S rRNA gene sequence analysis.** Total DNA was extracted from fecal samples using a DNA Microprep Kit (ZymoBIOMICS) according to the recommendations of the manufacturer. The extracted DNA was then used as a template to amplify the V4 regions of 16S rRNA genes using the universal primers 515F (5′-GTGCCAGCMGCCGCGGTAA-3′) and 806R (5′-GGACTACHVGGGTWTCTAAT-3′). Each reaction contained 2x PCR buffer, 1.5 mM $MgCl_2$, 0.4 μM dNTPs, 0.5 U KOD-Plus-Neo (Toyobo), 1.0 μM of each primer, and 10 ng of template DNA. The following cycling conditions were used: 94°C for 1 min, followed by 30 cycles at 94°C for 20 s, 55°C for 30 s and 72°C for 30 s, where the final extension step was performed at 72°C for 5 min. Replicate PCRs were combined and were subsequently purified using the OMEGA Gel Extraction Kit (Omega Bio-Tek, U.S.A.). Using the Qubit 2.0 fluorometer (Thermo Fisher Scientific, U.S.A.), PCR products were quantified and pooled at equimolar amounts. The completed library was sequenced using an Illumina HiSeq system with a HiSeq Rapid SBS Kit V2 (Illumina, U.S.A.). The sequences were analyzed according to Usearch and QIIME pipelines.[8] Paired-end reads from the original DNA fragments were merged using FLASH.[9] The sequences were clustered into Operational Taxonomic Units (OTUs) using the UPARSE algorithm[10] with a 97% threshold of pairwise identity and classified taxonomically using the Silva database[11] and Uclust classifier in QIIME. Non-metric Multi-Dimensional Scaling (NMDS) was performed using Vegan.

**Short-chain fatty acids analysis.** Quantification analysis of fecal short-chain fatty acids (SCFAs), including acetic acid, propionic acid, butyric acid, isobutyric acid, valeric acid, isovaleric acid, and hexanoic acid, were performed on an Agilent 7890A gas chromatography equipped with an Agilent 5975C mass spectrometric detector. To this end, 0.1 g of fecal samples was added to a microfuge tube containing 1000 μL of 0.5% $H_3PO_4$, then homogenized for 2 min, centrifuged at 17949g for 10 min. Then 800 μL of supernatant was extracted with equal amounts of ethyl acetate and allowed to vortex for 2 min. Samples were centrifuged at 17949g for 10 min. The internal standard of 4-methylpentanoic acid was spiked into the supernatant, and transferred to a gas chromatograph (GC) vial. Concentrations of individual SCFAs were analyzed with a polar DB-WAX capillary column (30 m × 0.25 mm × 0.25 μm, Agilent, CA). Helium was used as a carrier gas at a constant flow rate of 1 mL/min. The injector temperature was 250°C and the ion source temperature was 230°C. The initial oven temperature was 90°C, rising to 120°C at 10°C/min, then to 150°C at 5°C/min, and finally to 250°C at a rate of 25°C/min, with a final hold at this temperature for 2 min.

**Therapeutic effects of Ac2-26 in mice with acute peritonitis.** Male BALB/c mice (18-20 g) were i.p. administered with 1 mg zymosan. At 1 h after zymosan injection, mice received i.p. injection of saline, free Ac2-26, ON, APN, or AON in 200 μL of sterile saline. For all Ac2-26 containing formulations, its dose was 500 ng Ac2-26 per mouse. At predetermined time points, peritoneal exudates were collected by lavaging with 5 mL of sterile saline. Peritoneal exudate cells were collected by centrifugation, stained with V450-conjugated rat anti-mouse CD11b antibody, PE-conjugated rat anti-mouse Ly6G antibody, or isotype controls, and then assessed via a BD FACSVerse flow cytometer. The levels of TNF-α, IL-1β, MPO, MDA, and hydrogen peroxide in peritoneal exudates were quantified using the corresponding assay kits. In brief, peritoneal exudates were collected, into which 500 μL of Triton X-100 (0.3 wt%) was added. The mixture was homogenized and centrifuged at 12000g for 10 min at 4°C. The manufacturer's instructions were followed for quantification.

**Acute toxicity evaluation of AON.** Female C57BL/6J mice were randomly divided into two groups ($n = 5$). AON in saline was orally administered at a dose of 5.0 g/kg. In the control group, mice were administered 0.2 mL of saline by oral gavage. After administration, the body weight of mice and their behaviors were monitored each day. After two weeks, animals were euthanized. Blood samples were collected for hematological analysis. Major organs including heart, liver, spleen, lung, kidney, stomach, intestine, and MLN were isolated and weighed for calculation of the organ index. Histopathological sections were prepared and stained with H&E.

**Statistical analysis.** Data are expressed as mean ± standard error of the mean (SE). Statistical analysis was assessed using One-way ANOVA test. A value of $P < 0.05$ was considered statistically significant.

**References**

[1] D. Zhang, Y. Wei, K. Chen, X. Zhang, X. Xu, Q. Shi, S. Han, X. Chen, H. Gong, X. Li, J. Zhang, *Adv. Healthc. Mater.* **2015**, *4*, 69.
[2] S. Wirtz, V. Popp, M. Kindermann, K. Gerlach, B. Weigmann, S. Fichtner-Feigl, M. F. Neurath, *Nat. Protoc.* **2017**, *12*, 1295.
[3] H. S. Cooper, S. N. Murthy, R. S. Shah, D. J. Sedergran, *Lab. Invest.* **1993**, *69*, 238.
[4] J. Zhu, Y. Wang, F. Yang, L. Sang, J. Zhai, S. Li, Y. Li, D. Wang, C. Lu, X. Sun, *Int. Immunopharmacol.* **2015**, *29*, 846.
[5] J. W. Heinecke, H. Rosen, A. Chait, *J. Clin. Invest.* **1984**, *74*, 1890.
[6] K. Takeda, B. E. Clausen, T. Kaisho, T. Tsujimura, N. Terada, I. Forster, S. Akira, *Immunity* **1999**, *10*, 39.
[7] J. S. Savill, A. H. Wyllie, J. E. Henson, M. J. Walport, P. M. Henson, C. Haslett, *J. Clin. Invest.* **1989**, *83*, 865.
[8] J. G. Caporaso, J. Kuczynski, J. Stombaugh, K. Bittinger, F. D. Bushman, E. K. Costello, N. Fierer, A. G. Peña, J. K. Goodrich, J. I. Gordon, G. A. Huttley, S. T. Kelley, D. Knights, J. E. Koenig, R. E. Ley, C. A. Lozupone, D. McDonald, B. D. Muegge, M. Pirrung, J. Reeder, J. R. Sevinsky, P. J. Turnbaugh, W. A. Walters, J. Widmann, T. Yatsunenko, J. Zaneveld, R. Knight, *Nat. Methods* **2010**, *7*, 335.
[9] T. Magoc, S. L. Salzberg, *Bioinformatics* **2011**, *27*, 2957.
[10] R. C. Edgar, *Nat. Methods* **2013**, *10*, 996.
[11] C. Quast, E. Pruesse, P. Yilmaz, J. Gerken, T. Schweer, P. Yarza, J. Peplies, F. O. Glockner, *Nucleic Acids Res.* **2013**, *41*, D590.

**Supplementary Results**

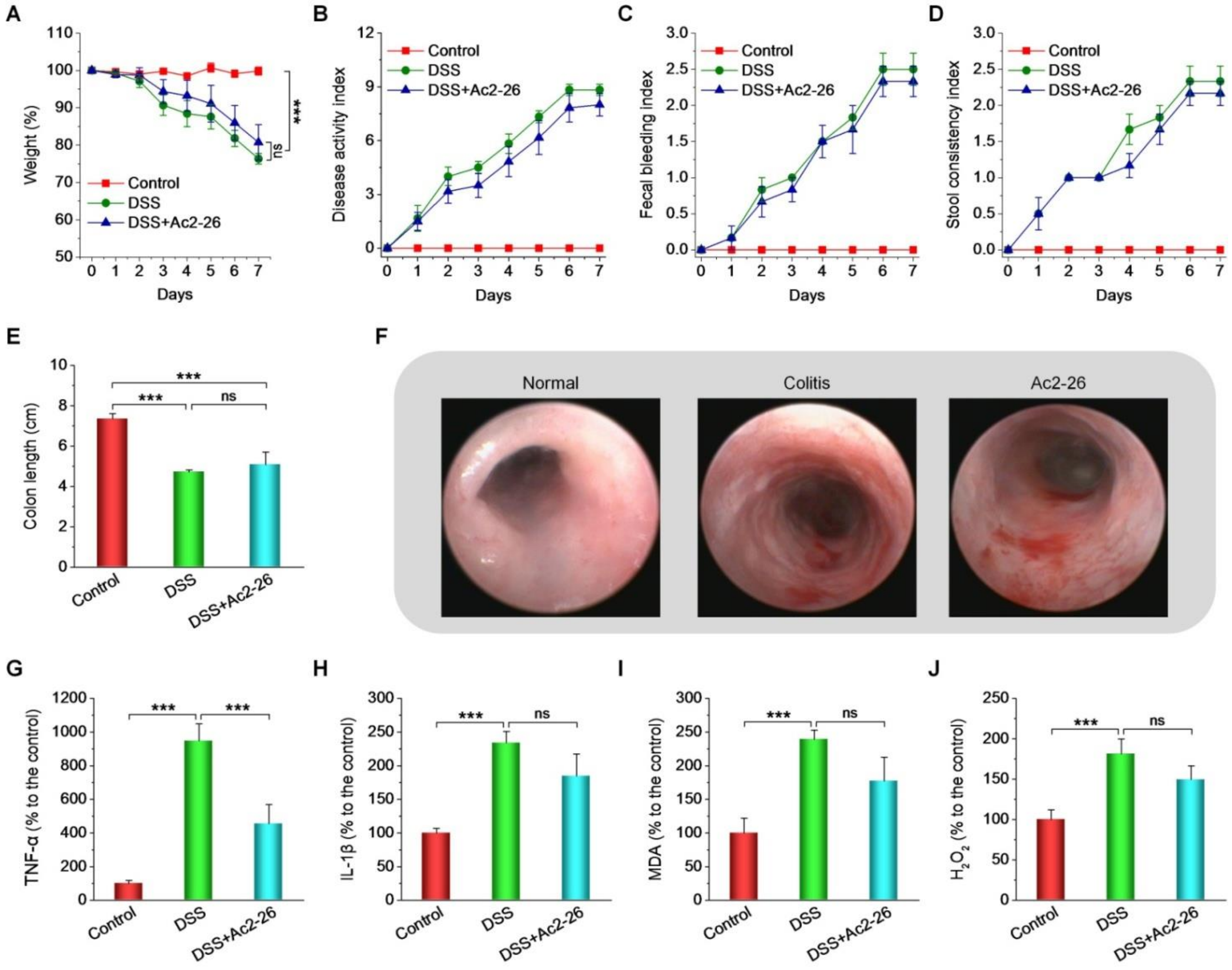


**Figure S1.** Therapeutic effects of free Ac2-26 on DSS-induced acute colitis in mice. (A) The body weight of mice during 7 days of treatment. Data were normalized as the percentage of body weight at day 0. Healthy mice in the normal group were not treated, while colitis mice were orally administered with saline or Ac2-26 (at 250 μg/kg) daily during 7 days of 3% DSS treatment. (B) Changes in disease activity index (DAI), which is the summation of the stool consistency index (0-3), fecal bleeding index (0-3), and weight loss index (0-4). (C-D) The fecal bleeding index (C) and stool consistency index (D) of mice with DSS colitis. (E) The length of colonic tissues isolated from mice after 7 days of treatment. (F) Representative mini-endoscopic images of colons from mice subjected to various treatments. (G-J) The levels of TNF-α (G), IL-1β (H), MDA (I), and $H_2O_2$ (J). After 7 days of treatment, homogenates of the colonic tissues were prepared, and the concentrations of various mediators were separately measured by commercial kits. The total protein was measured by the BCA assay. Data are presented as mean ± SE ($n$ = 6). $^{*}P < 0.05$, $^{**}P < 0.01$, $^{***}P < 0.001$; ns, no significance.

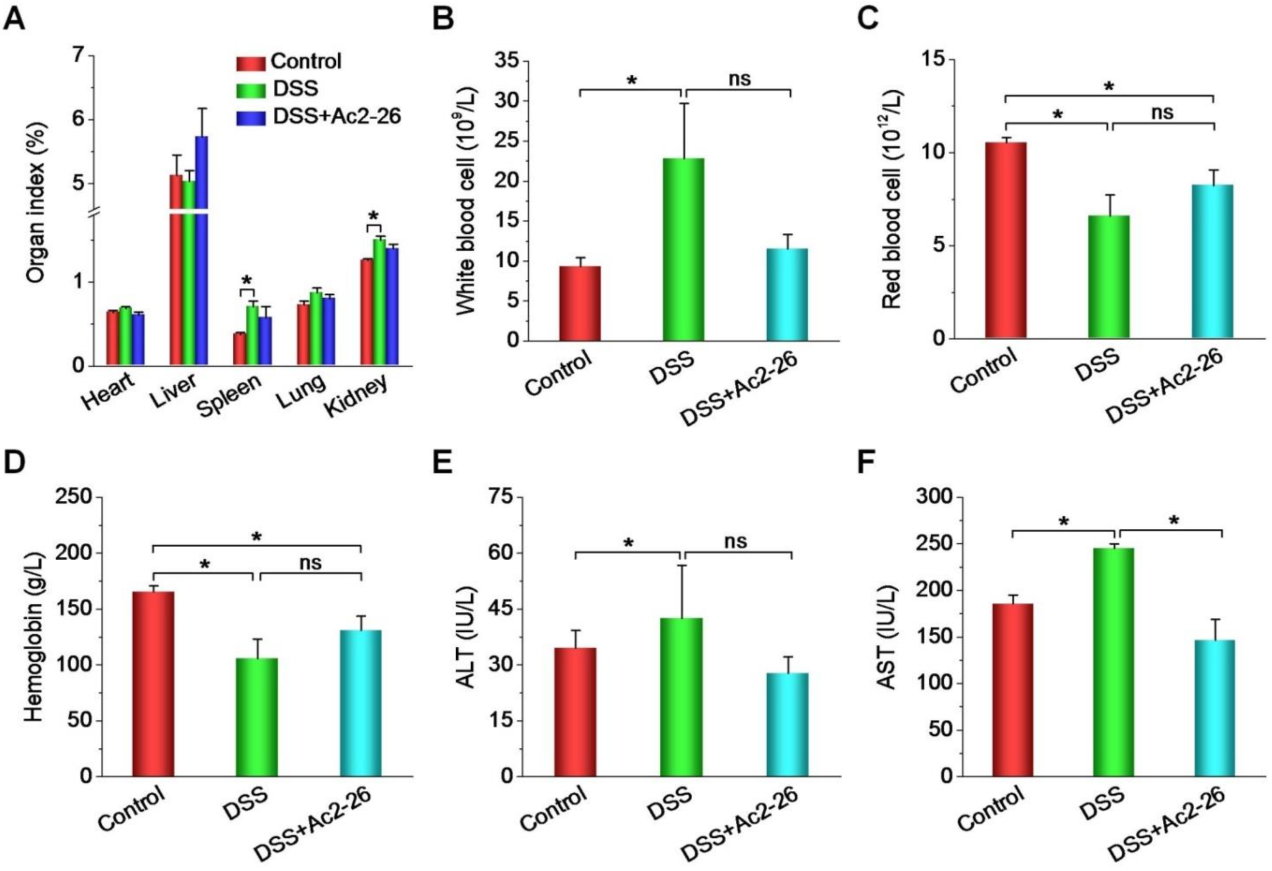


**Figure S2.** Evaluations of in vivo therapeutic benefits after treatment with Ac2-26. (A) The organ index of major organs isolated from mice subjected to different treatments after induction with 3% DSS. (B-D) White blood cell (B), red blood cell (C), and hemoglobin (D). (E-F) The levels of ALT (E) and AST (F). ALT, alanine aminotransferase; AST, aspartate aminotransferase. Data are presented as mean ± SE ($n$ = 6). $^{*}P < 0.05$.

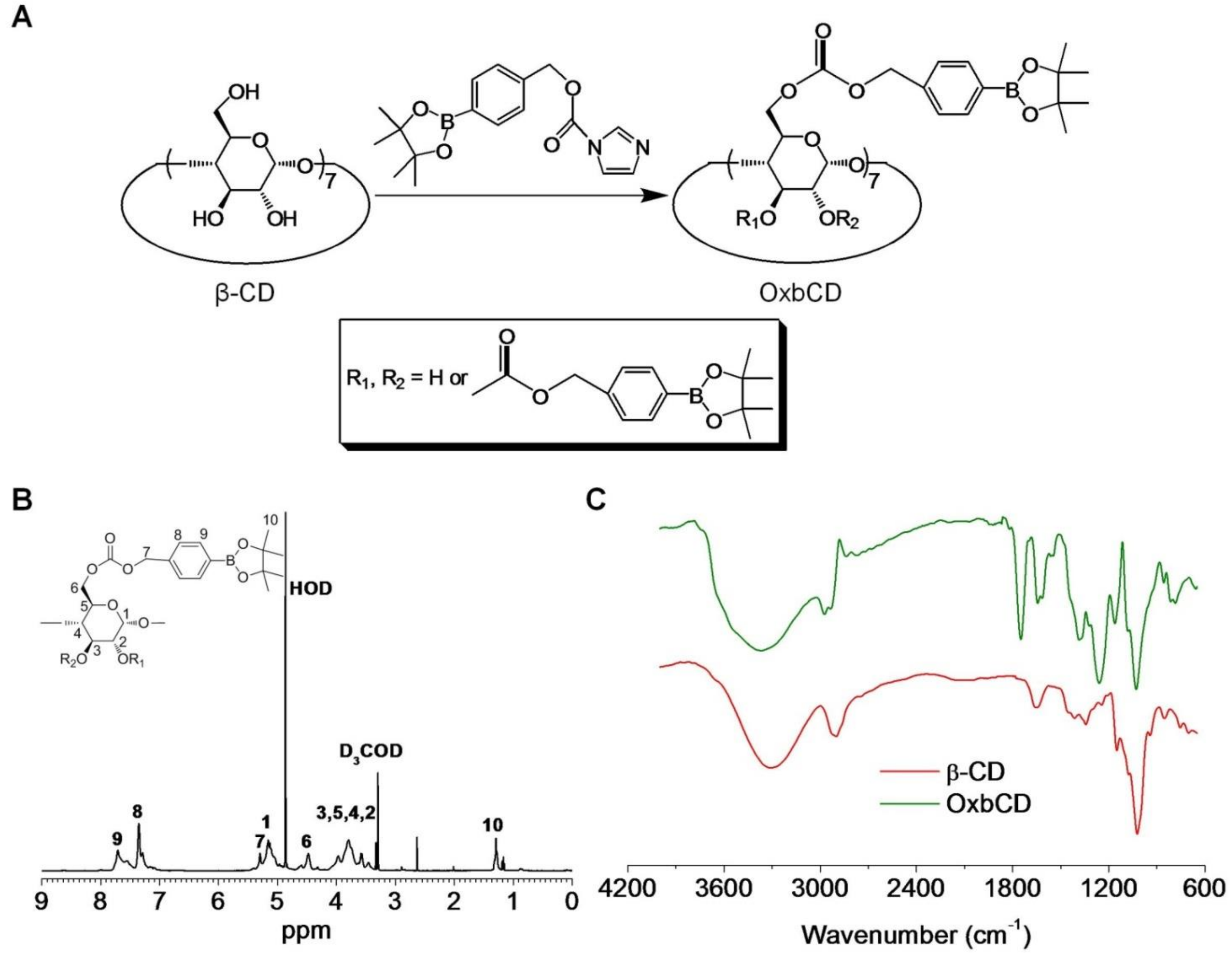


**Figure S3.** Synthesis and characterization of a ROS-responsive material OxbCD. (A) Schematic illustration of synthesis of OxbCD from a cyclic oligosaccharide β-cyclodextrin (β-CD). (B-C) Characterization of OxbCD by $^1$H NMR (B) and FT-IR (C) spectroscopy. In the image (B), R1 and R2 represent the groups of -H or 4-(acyloxymethyl) phenylboronic acid pinacol ester as indicated in the image (A).

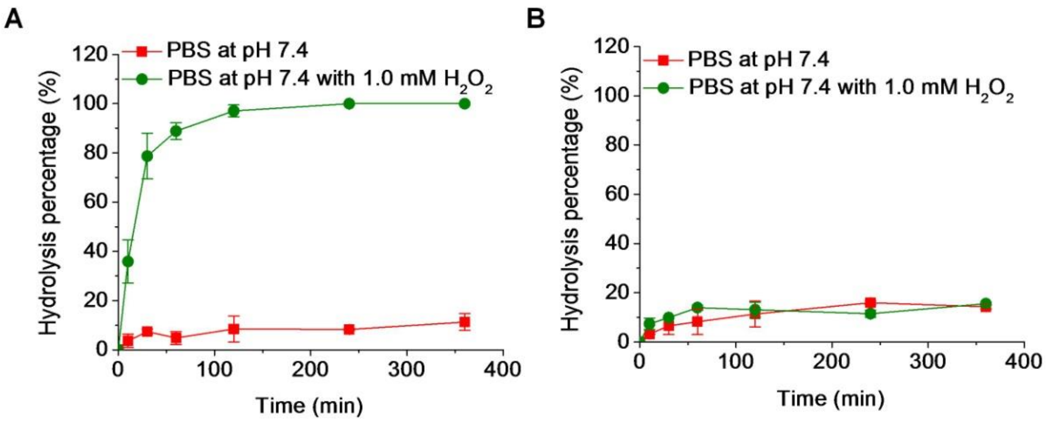


**Figure S4.** Hydrolysis profiles of different nanotherapies. (A-B) Hydrolysis curves of AON (A) or APN (B) in PBS with or without 1 mM $H_2O_2$. Data are presented as mean ± SE ($n$ = 3).

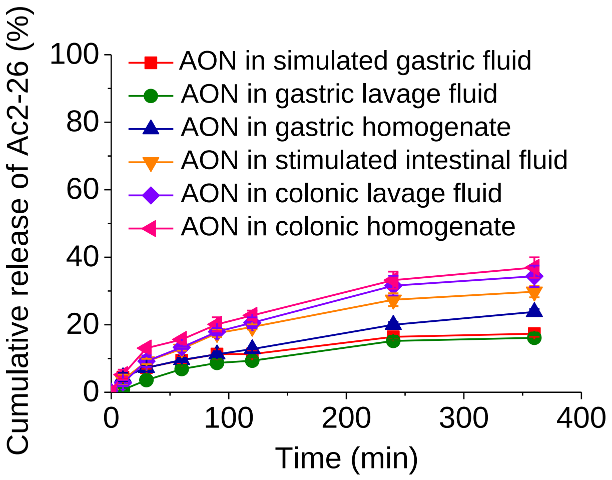


**Figure S5.** In vitro release profiles of AON in different simulated gastrointestinal fluids. Data are presented as mean ± SE (*n* = 3).

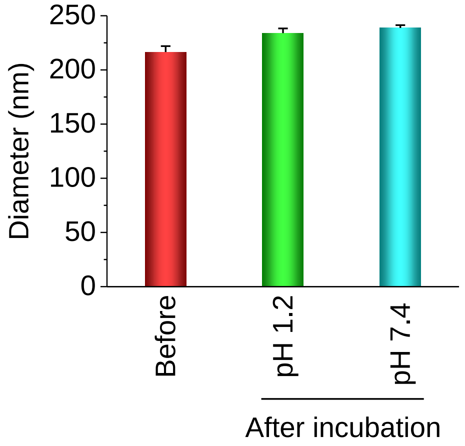


**Figure S6.** Mean hydrodynamic diameters of AON before and after incubation in simulated gastric fluid for 2 h (pH 1.2) and then incubated in simulated intestinal fluid for 4 h (pH 7.4). Data are presented as mean ± SE (*n* = 3).

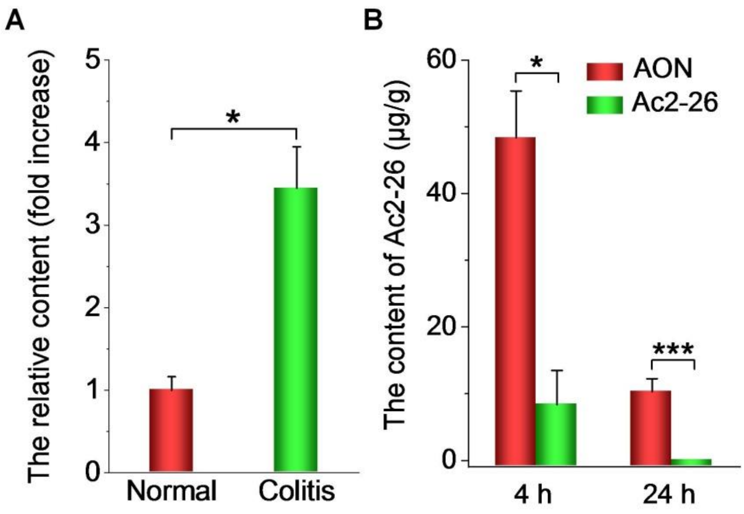


**Figure S7.** Quantification of the distribution of Ac2-26 in colonic tissues by HPLC. (A) The relative levels of Ac2-26 in the colons of mice with or without DSS-induced acute colitis at 4 h after oral administration of AON. (B) The contents of Ac2-26 in the colonic tissues from colitis mice at 4 and 24 h after treatment with free Ac2-26 or AON. In both cases, the dose was 50 μg of Ac2-26 for each mouse. Data are presented as mean ± SE ($n$ = 3). $^{*}P < 0.05$, $^{***}P < 0.001$.

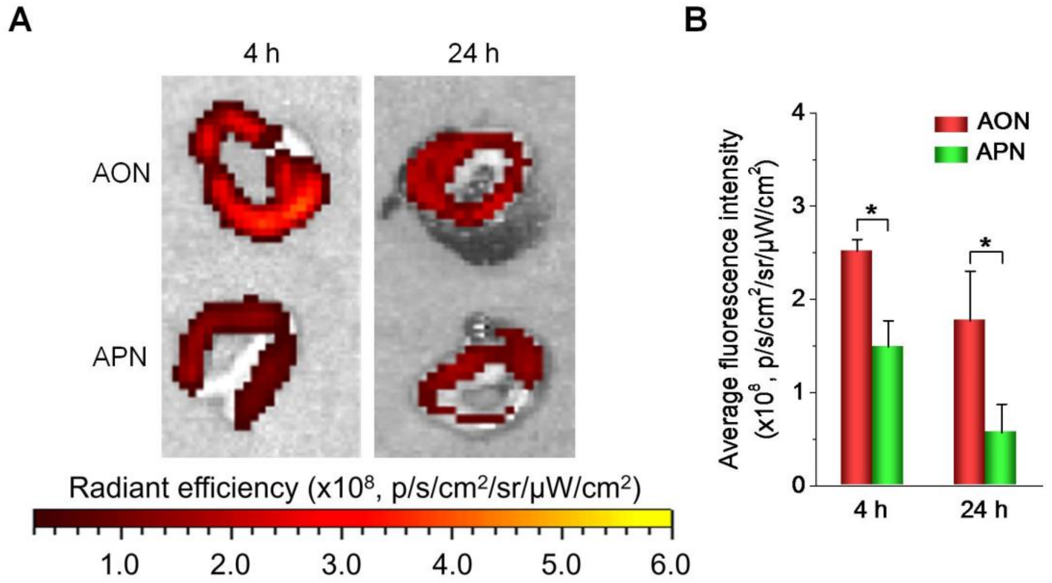


**Figure S8.** Quantification of the colon tissue distribution after treatment with two nanotherapies. (A-B) Ex vivo images (A) and quantitative data (B) illustrating the fluroescence intensities in the colonic tissue from colitis mice separately treated with Cy7.5-AON or Cy7.5-APN. Data are presented as mean ± SE ($n$ = 3). $^{*}P < 0.05$.

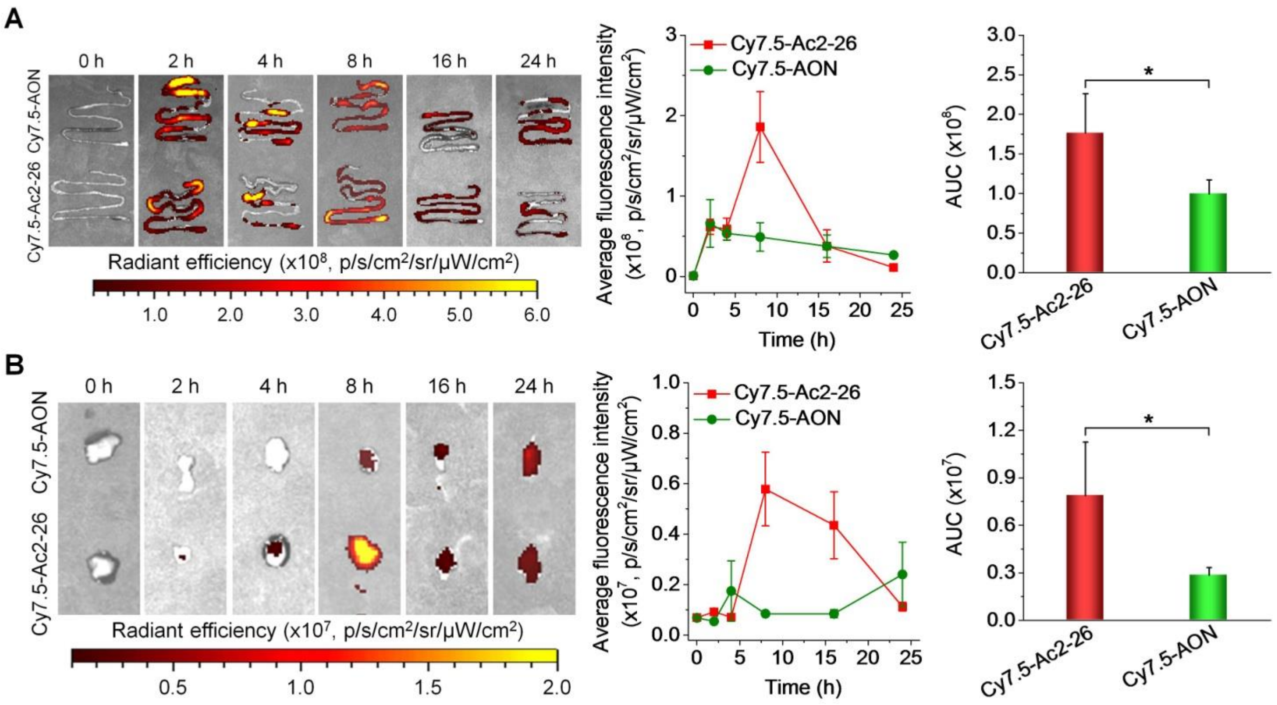


**Figure S9.** Ex vivo imaging of the tissue distribution of orally administered free Ac2-26 or AON at different time points in mice with acute colitis. (A-B) Ex vivo images (left), quantitative data (middle), and AUC values (right) of the intestine (A) and mesenteric lymph nodes (MLNs) (B). After 7 days of treatment with 3% DSS, mice received a single oral administration of free Cy7.5-Ac2-26 or Cy7.5-AON (containing 0.04 mg of Cy7.5). The colon segments and MLNs were isolated. Ex vivo imaging was carried out with a living imaging system. Data are presented as mean ± SE ($n$ = 3). $^{*}P < 0.05$.

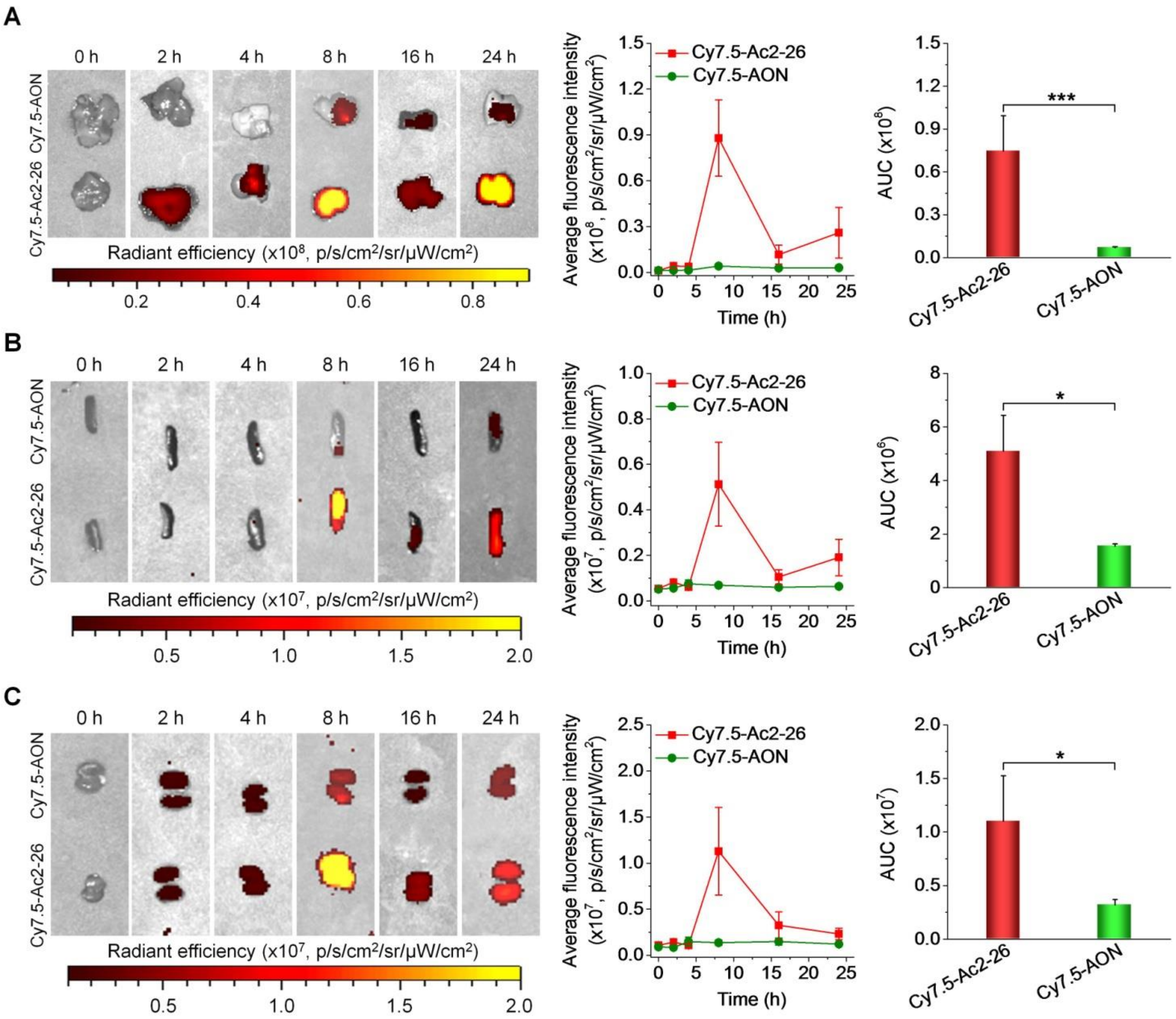


**Figure S10.** Ex vivo imaging of the tissue distribution of orally administered Ac2-26 or AON at different time points in colitis mice. (A-C) Ex vivo images (left), quantitative data (middle), and AUC values (right) of liver (A), spleen (B), and kidney (C). After 7 days of treatment with 3% DSS, mice received a single oral administration of free Cy7.5-Ac2-26 or Cy7.5-AON (containing 0.04 mg of Cy7.5). The major organs including liver, spleen, and kidney were isolated. Ex vivo imaging was carried out with a living imaging system. Data are presented as mean ± SE ($n$ = 3). *$P$ < 0.05, ***$P$ < 0.001.

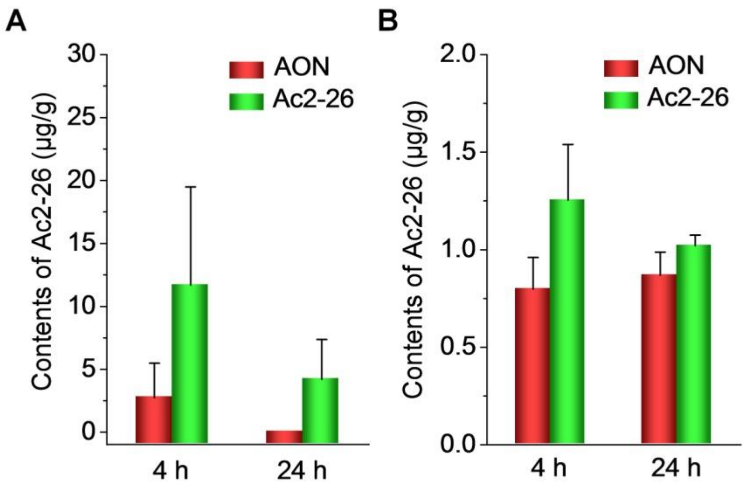


**Figure S11.** Quantification of Ac2-26 distribution in the spleen and liver. (A-B) The contents of Ac2-26 in the spleen (C) and liver (D) quantified by HPLC. After 7 days of treatment with 3% DSS, mice received a single oral administration of free Ac2-26 or AON at 50 μg Ac2-26 for each mouse. Ac2-26 concentrations in the spleen and liver were quantified by HPLC. Data are presented as mean ± SE ($n$ = 3).

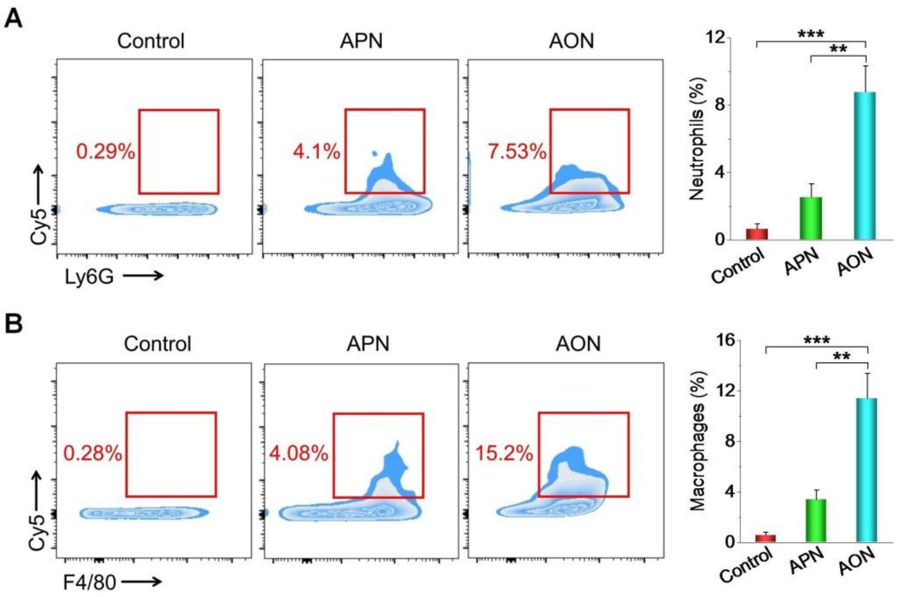


**Figure S12.** Quantification of the cellular distribution after treatment with two nanotherapies. (A-B) The distribution of Cy5-AON and Cy5-APN in neutrophils (A) and macrophages (B). The left panels show representative flow cytometry contour plots, while the right panels show corresponding quantitative data. Data are presented as mean ± SE ($n$ = 3). $^{**}P < 0.01$, $^{***}P < 0.001$.

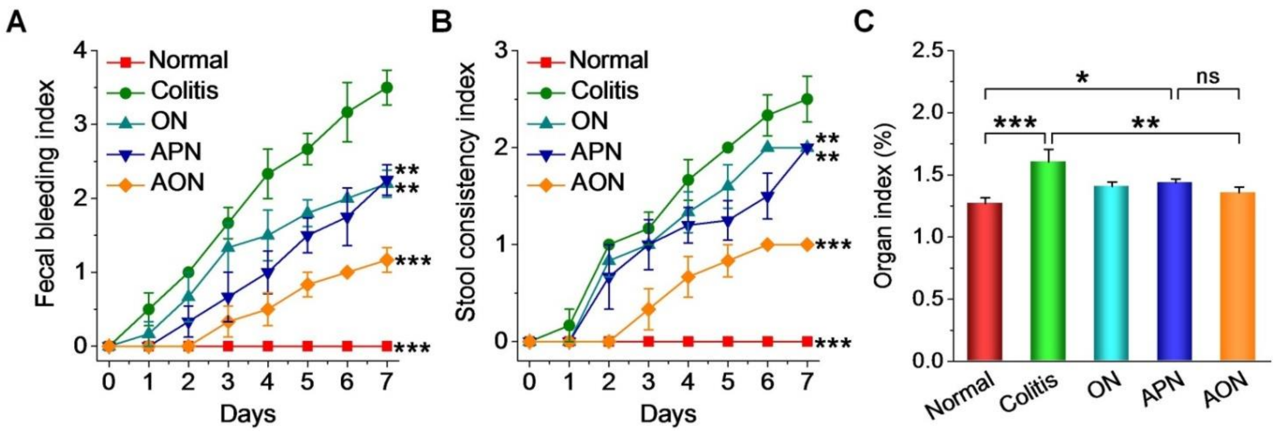


**Figure S13.** The effects of various treatments on typical indexes relevant to acute colitis in mice. (A-B) The fecal bleeding index (A) and stool consistency index (B) of mice with acute colitis. $^{**}P <$ 0.01 and $^{***}P < 0.001$ versus the colitis group. (C) The organ index of kidneys isolated from mice subjected to different treatments in DSS-induced colitis mice. Healthy mice in the normal group were not treated, while colitis mice were orally administered with saline, ON, APN, and AON, respectively. For all diseased mice, daily administration was performed during 7 days of 3% DSS treatment. The dose was 250 μg/kg of Ac2-26 for all Ac2-26-containing formations. All data are expressed as mean ± SE ($n$ = 6). For data in (C), $^{*}P < 0.05$, $^{**}P < 0.01$, $^{***}P < 0.001$; ns, no significance.

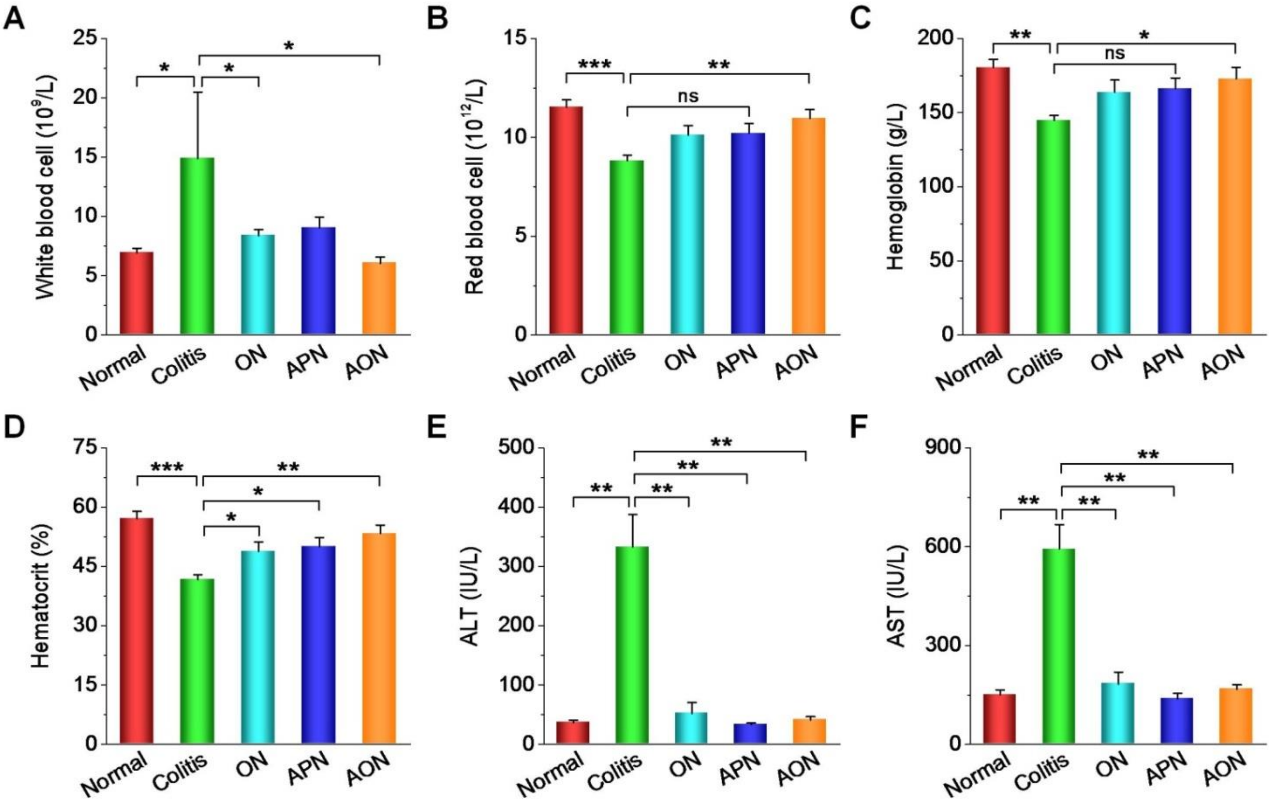


**Figure S14.** Typical hematological parameters and biochemical markers relevant to liver and kidney functions after various treatments in mice with acute colitis. (A-D) White blood cell (A), red blood cell (B), hemoglobin (C), and hematocrit (D). (E-F) The levels of ALT (E) and AST (F). Data are presented as mean ± SE ($n$ = 6). $^{*}P < 0.05$, $^{**}P < 0.01$, $^{***}P < 0.001$; ns, no significance.

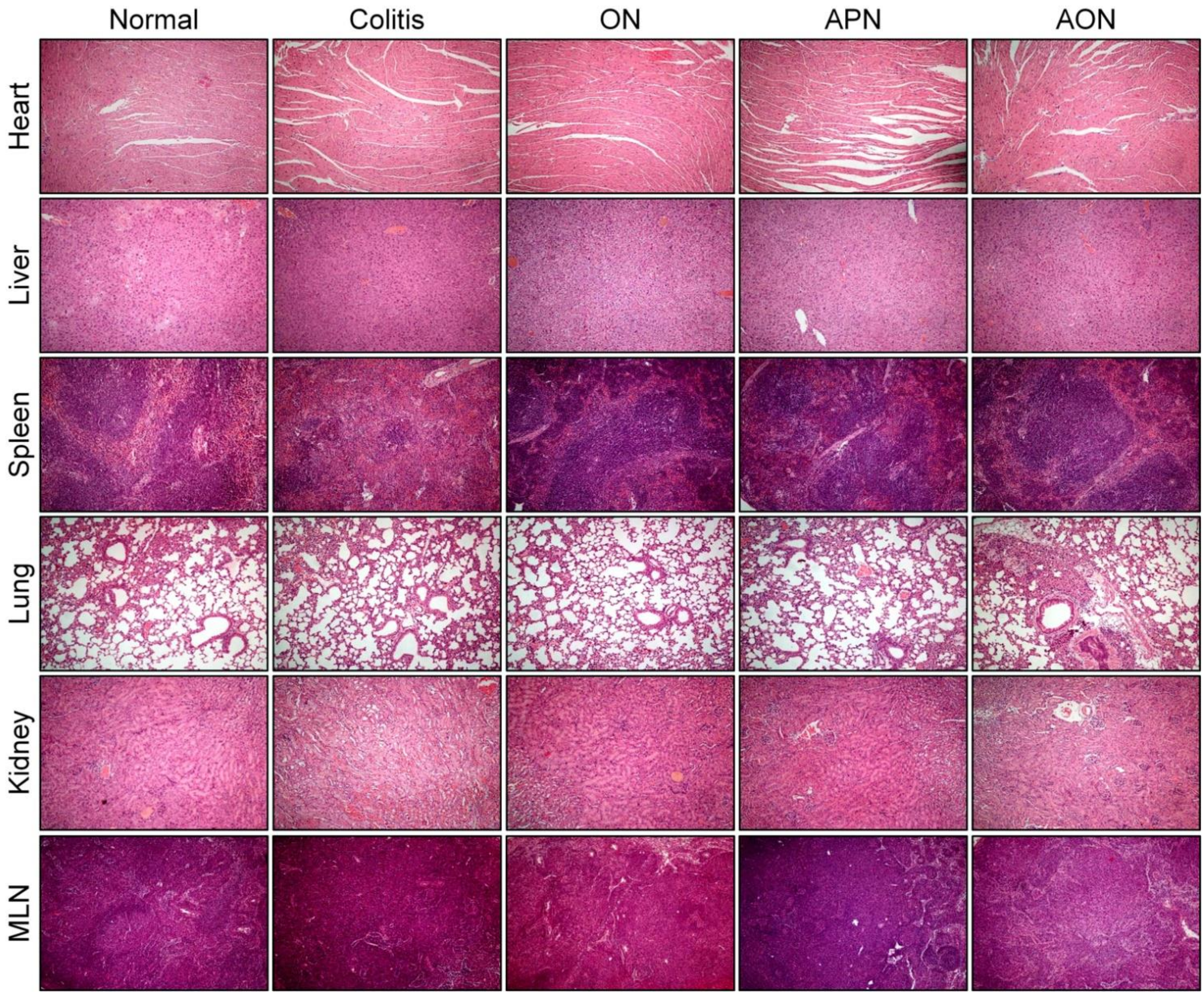


**Figure S15.** H&E-stained histological sections of representative major organs from mice with DSS-induced acute colitis after treatment with different formulations.

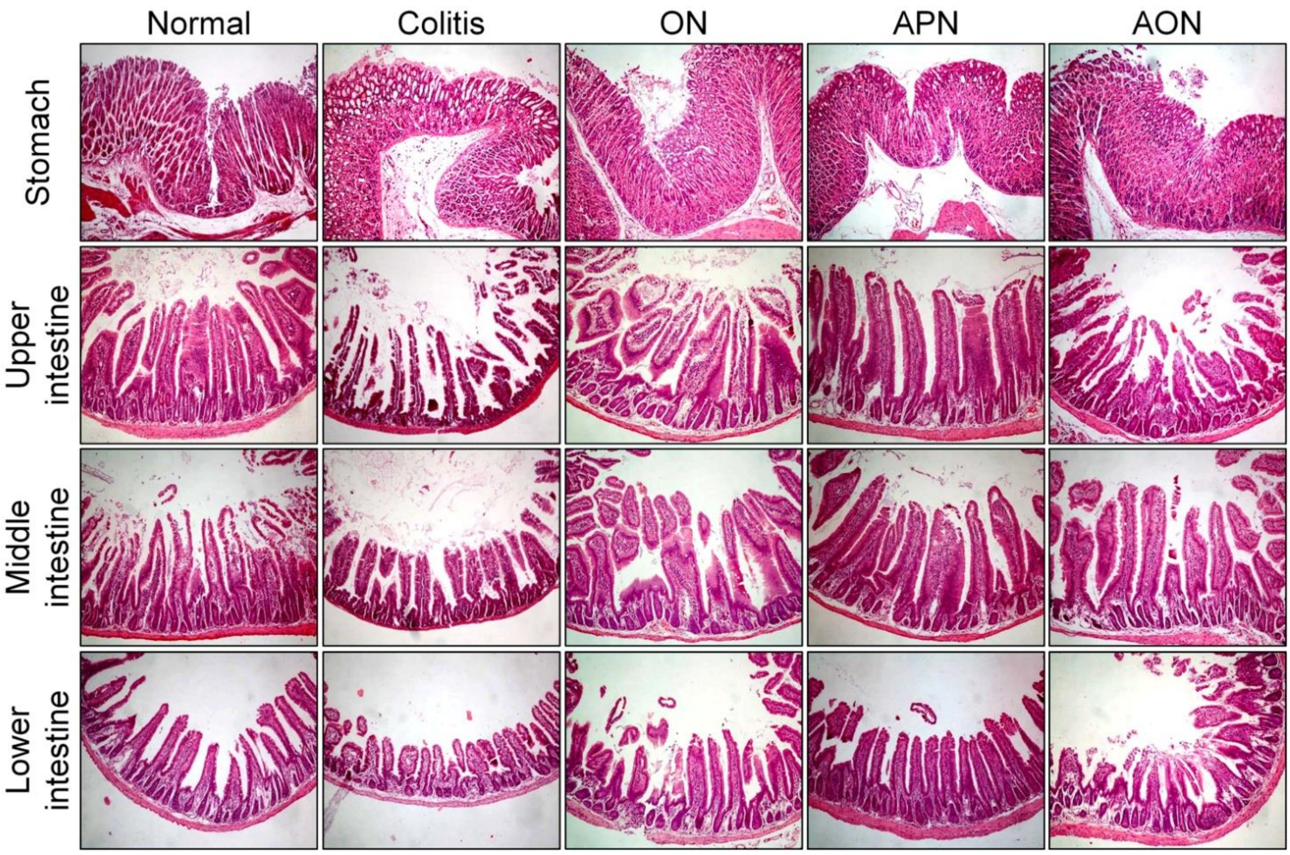


**Figure S16.** H&E-stained histological sections of gastrointestinal tissues from mice with DSS-induced acute colitis after treatment with different formulations.

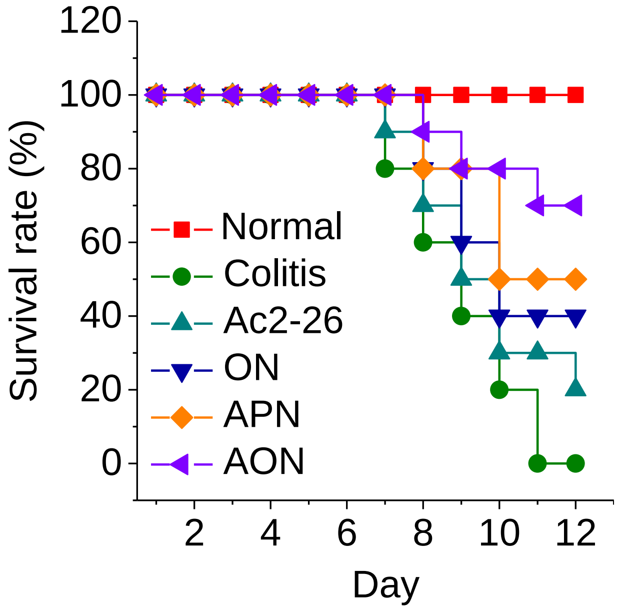


**Figure S17.** The survival rate of mice received different treatments. Mice in the normal group were treated with saline, while mice in other groups were treated with 3% DSS for 12 days. At day 1, different formulations were orally administered each day until day 12. The number of surviving mice was counted on day 12. For each group, 10 mice were used.

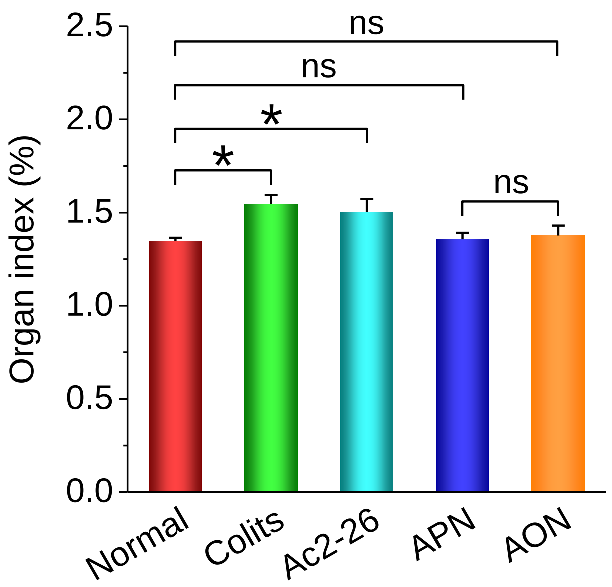


**Figure S18.** The organ index of kidneys isolated from mice with DSS-induced chronic colitis and subjected to different treatments. To induce chronic colitis in mice, animals were administered with 1.5% DSS in their drinking water ad libitum for three consecutive 5-day periods: days 1-5, 11-15, and 21-25. After each period of DSS administration, the animals were treated with saline, Ac2-26, APN, or AON by oral gavage. The healthy mice in the control group were allowed to drink water. The dose was 250 µg/kg of Ac2-26 for all Ac2-26-containing formations. Data are presented as mean ± SE ($n$ = 6). $^{*}P < 0.05$; ns, no significance.

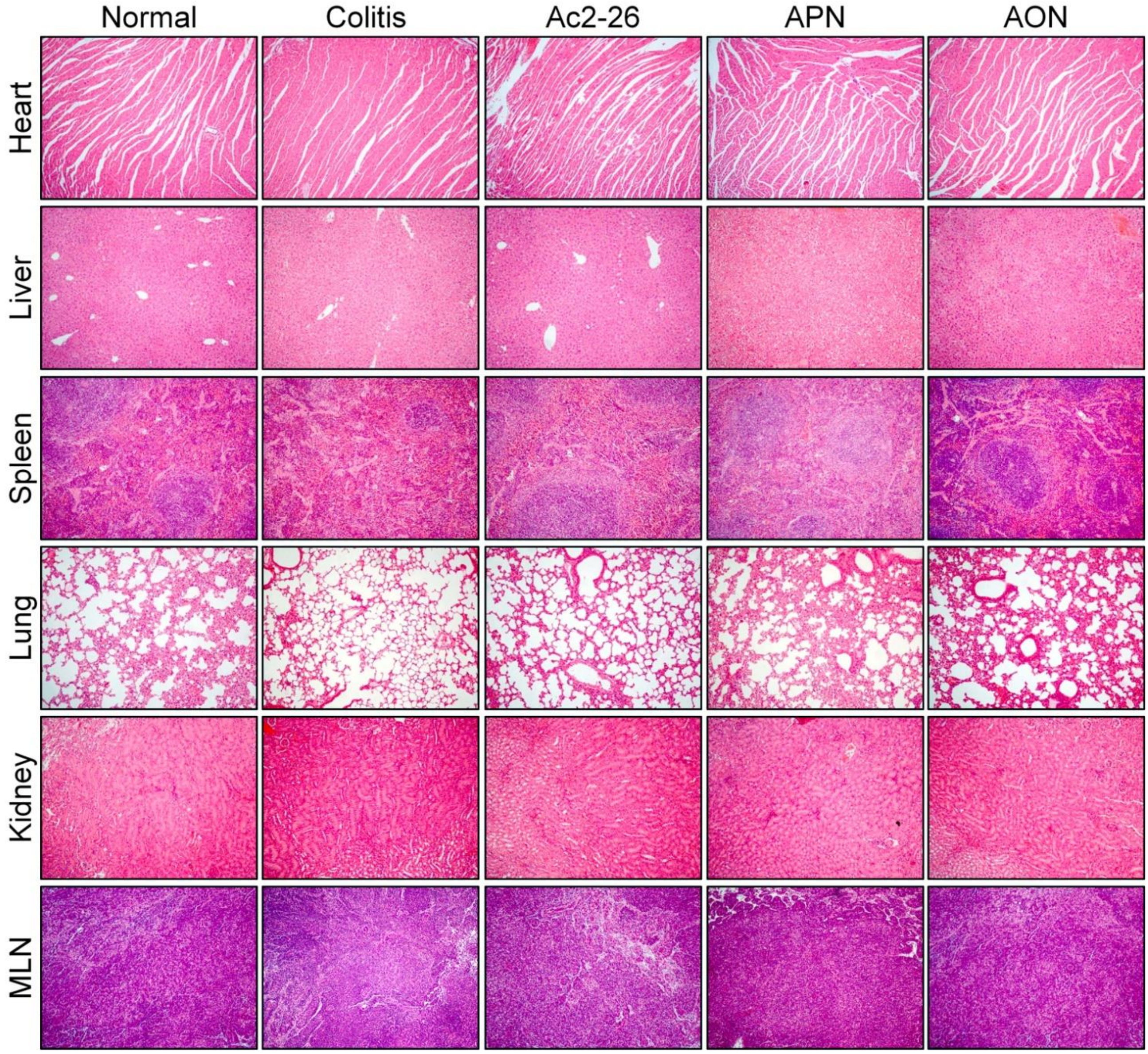


**Figure S19.** H&E-stained histological sections of representative major organs from mice with DSS-induced chronic colitis and after treatment with different formulations.

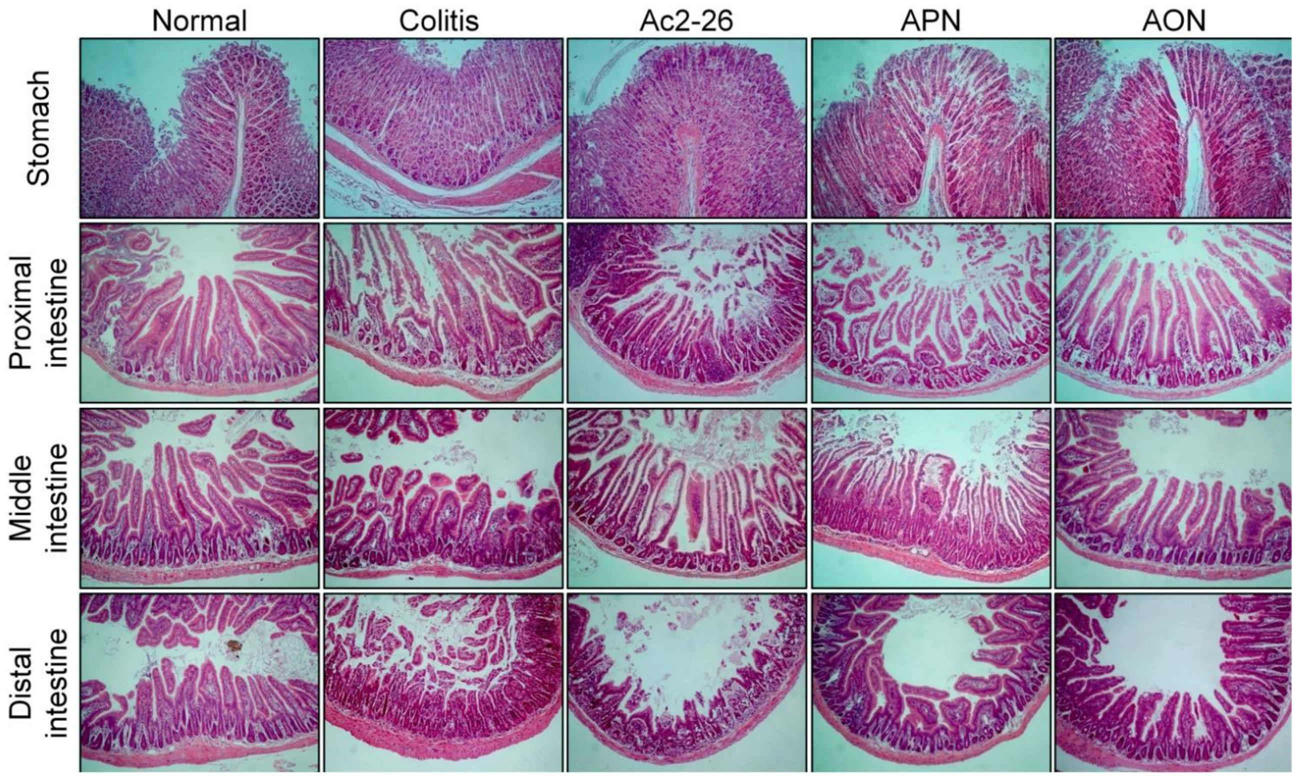


**Figure S20.** H&E-stained histological sections of gastrointestinal tissues from mice with DSS-induced chronic colitis and after treatment with different formulations.

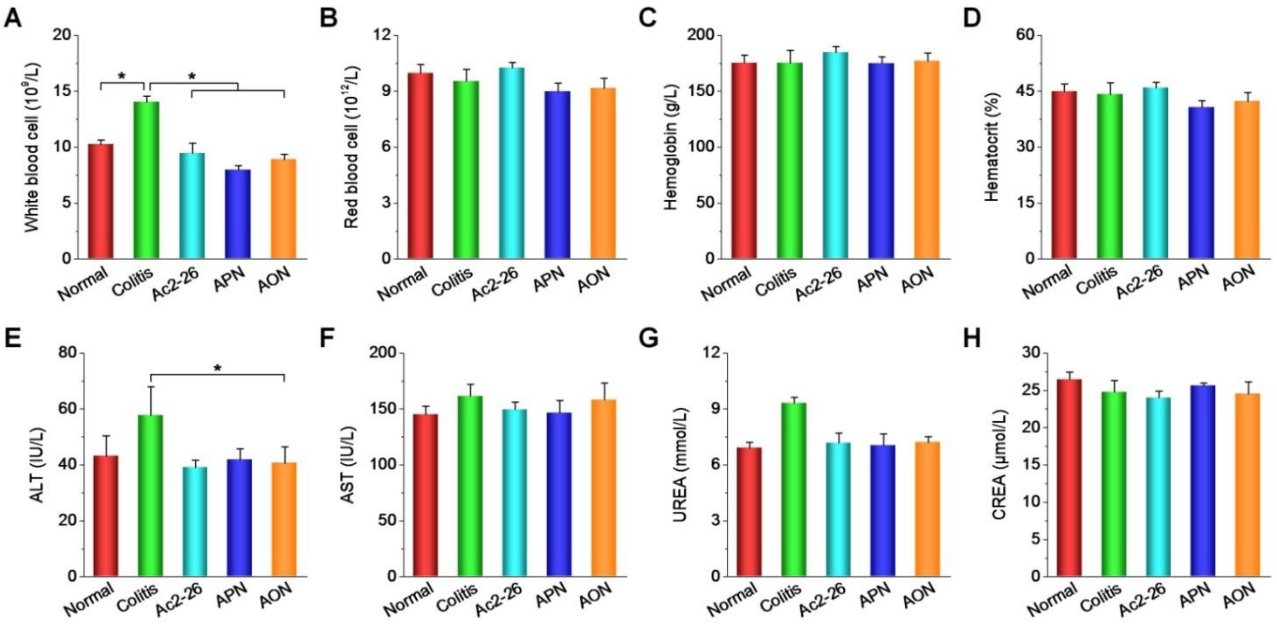


**Figure S21.** Safety evaluations of different nanotherapies after treatment of mice with induced chronic colitis. (A-D) White blood cell (A), red blood cell (B), hemoglobin (C), and hematocrit (D). (E-H) The levels of ALT (E), AST (F), UREA (G), and CREA (H). All data are expressed as mean ± SE ($n$ = 6). $^{*}P < 0.05$.

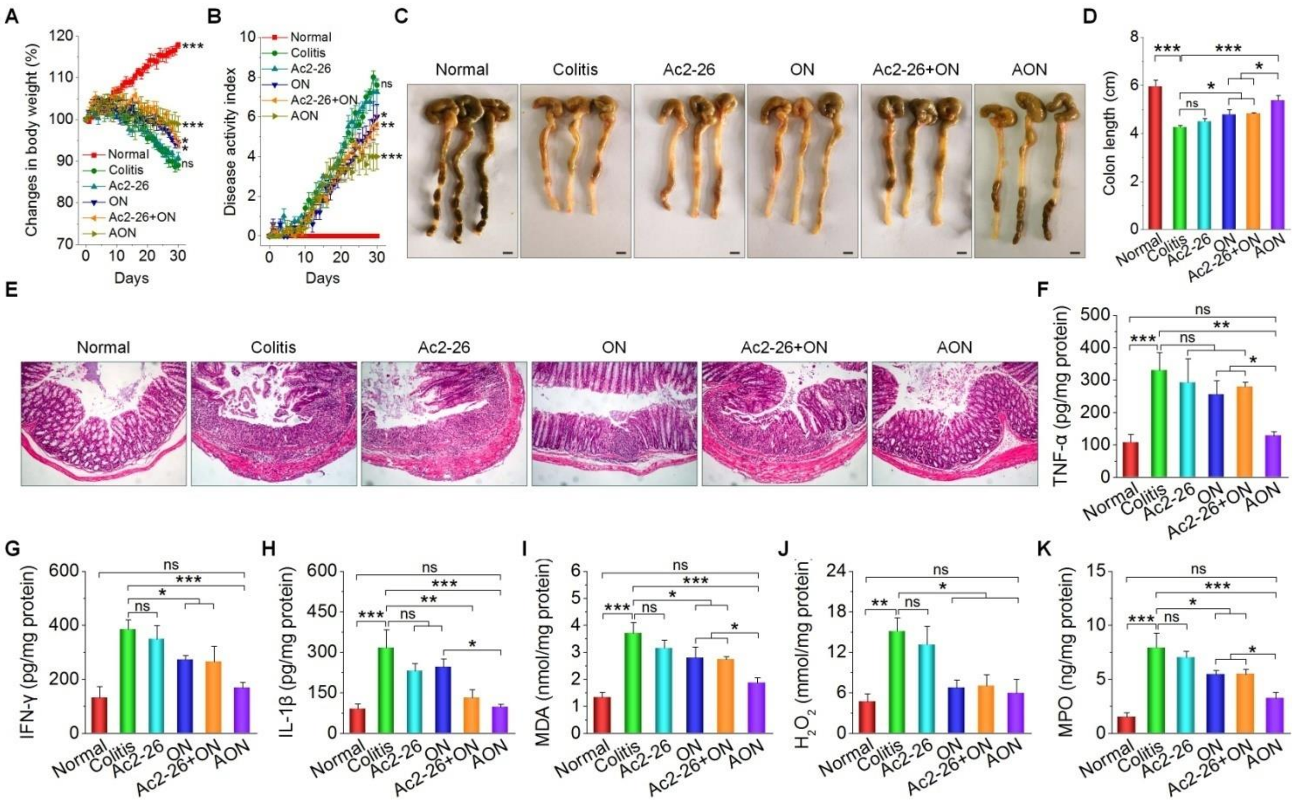


**Figure S22.** Comparison of therapeutic effects of AON with other formulations in mice with DSS-induced chronic colitis. (A-B) Changes in the mouse body weight (A) and DAI (B) during 30 days of treatment. $^{*}P < 0.05$, $^{**}P < 0.01$, $^{***}P < 0.001$, and ns (no significance) versus the colitis group. (C-E) Representative digital photos of colons (C), the measured colon length (D), H&E-stained histological sections of colonic tissues (E). Scale bars, 5 mm. (F-K) The levels of TNF-α (F), IFN-γ (G), IL-1β (H), MDA (I), $H_2O_2$ (J), and MPO (K) in colonic tissues. For data in (D, F-K), $^{*}P < 0.05$, $^{**}P < 0.01$, $^{***}P < 0.001$; ns, no significance. All data are presented as mean ± SE ($n$ = 6).

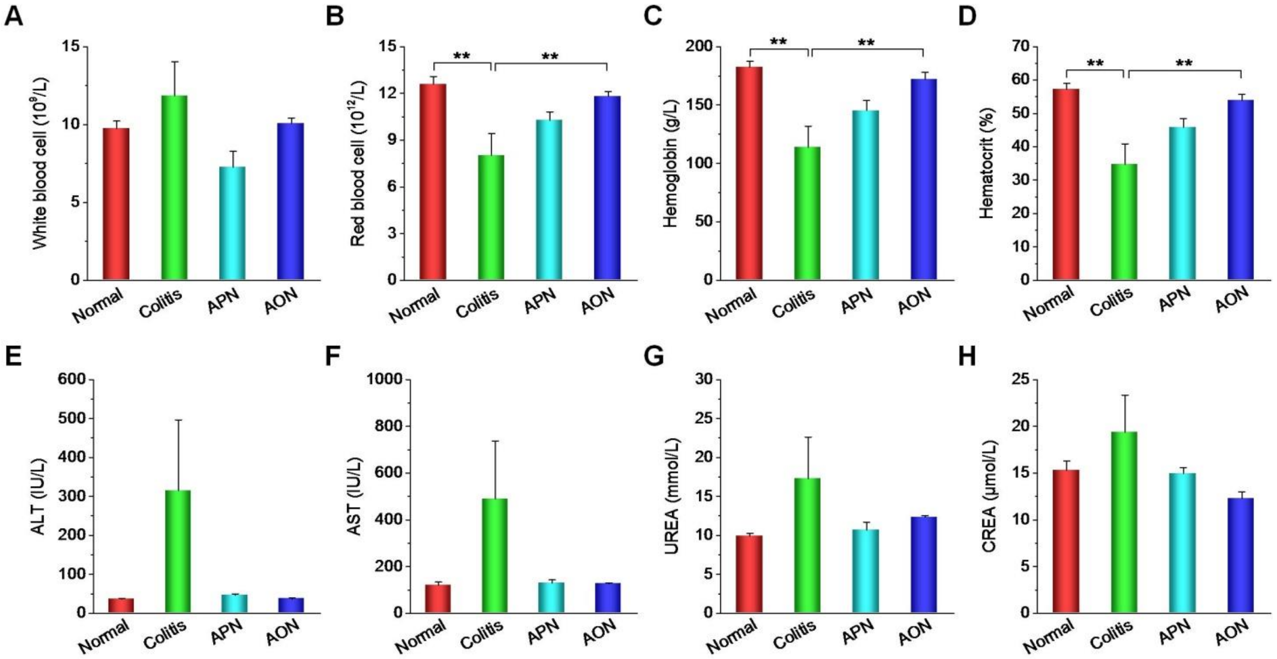


**Figure S23.** Safety evaluations of two nanotherapies after treatment of IL-$10^{-/-}$ mice with spontaneous chronic colitis. (A-D) White blood cell (A), red blood cell (B), hemoglobin (C), and hematocrit (D). (E-H) The levels of ALT (E), AST (F), UREA (G), and CREA (H). Data are expressed as mean ± SE ($n$ = 6). $^{**}P < 0.01$.

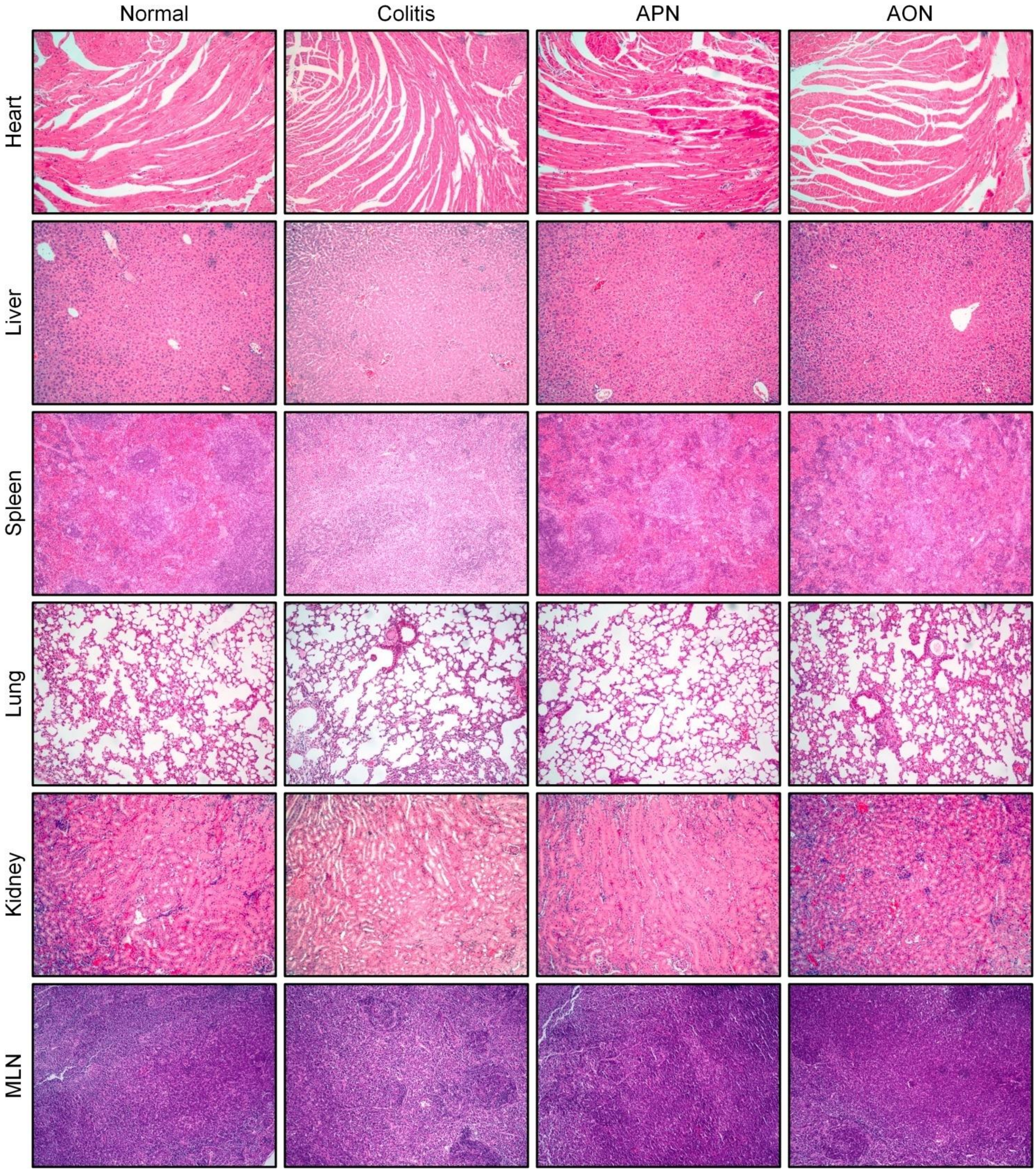


**Figure S24.** H&E-stained histological sections of major organs from IL-10$^{-/-}$ mice with spontaneous chronic colitisis and after treatment with different formulations.

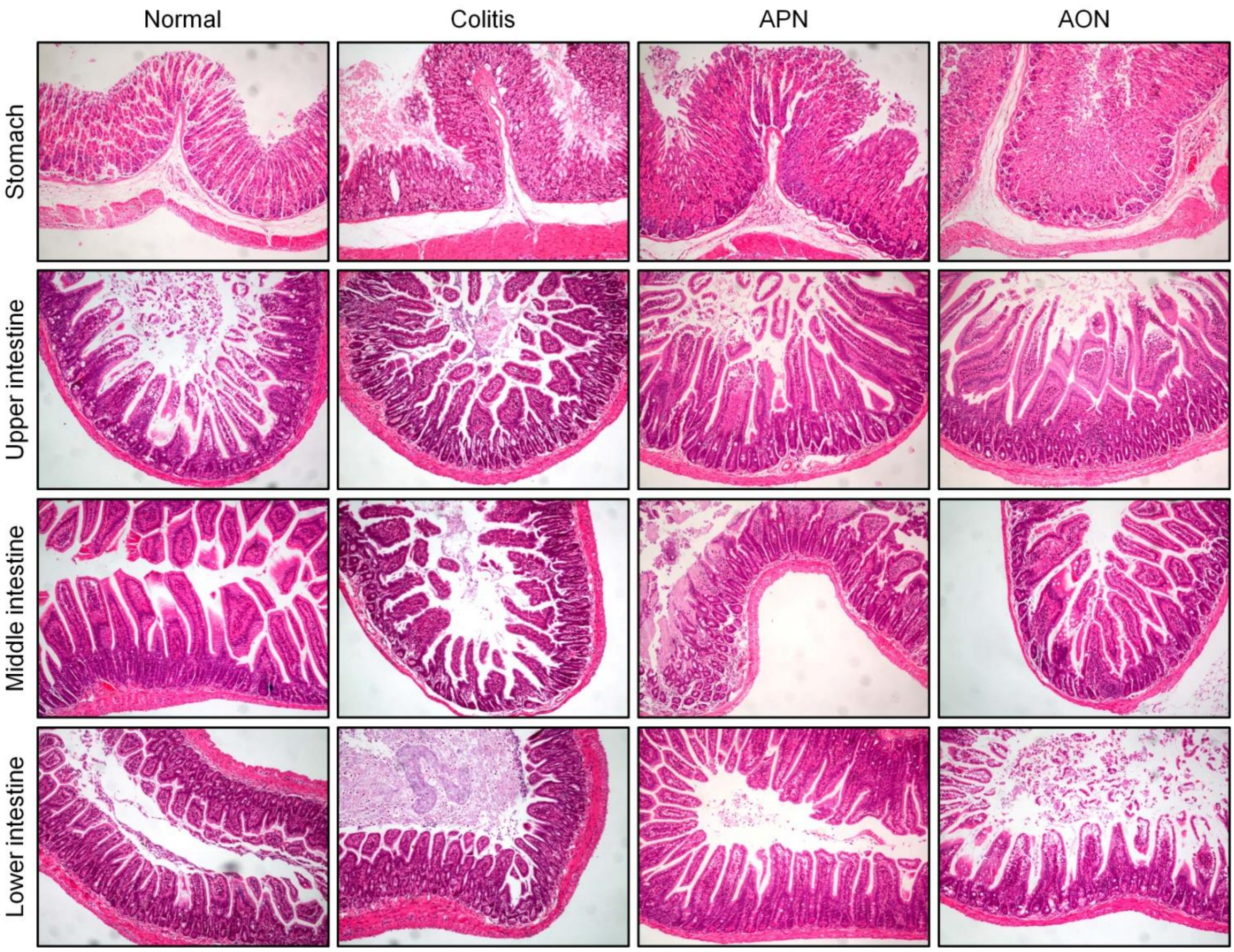


**Figure S25.** H&E-stained histological sections of gastrointestinal tissues from IL-10$^{-/-}$ mice with spontaneous chronic colitisis and after treatment with different formulations.

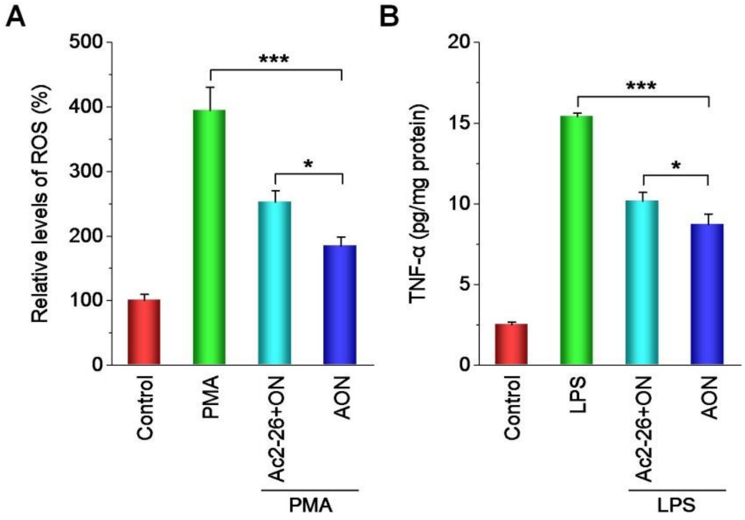


**Figure S26.** In vitro evaluation of antioxidative and anti-inflammatory activities. (A) The relative ROS levels in macrophages quantified by flow cytometry. Cells were stimulated with 100 ng/mL of PMA and then treated with the Ac2-26/ON mixture or AON for 4 h. DCFH-DA was used as a fluorescent probe to stain intracellular ROS. (B) The expression levels of TNF-α in macrophages. Macrophages were pre-incubated with the Ac2-26/ON mixture or AON for 6 h, and then exposed to 100 ng/mL of LPS for 12 h. The TNF-α concentration in the cell culture supernatant of was quantified by using a commercial ELISA kit.

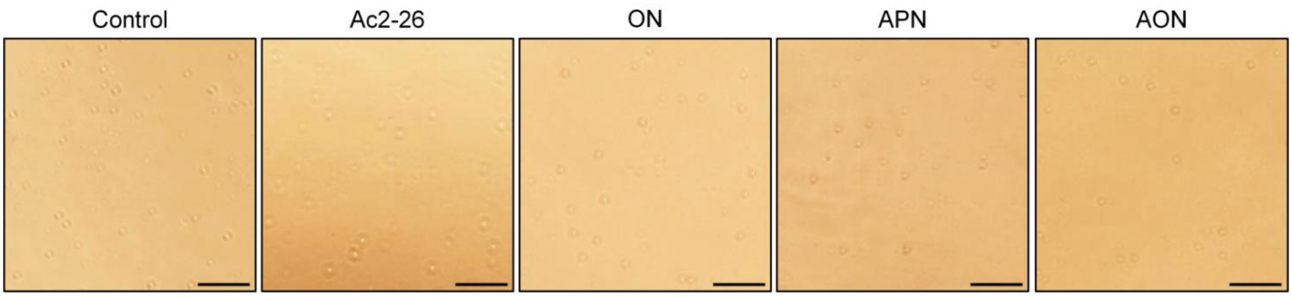


**Figure S27.** Optical microscopic images showing migrated neutrophils in the lower chamber of the transwell system after treatment with different formulations. Neutrophils were separately exposed to vehicle (the control group), Ac2-26, ON, APN, or AON on the upper chamber of the transwell system. The dose of Ac2-26 was 1 μM for different Ac2-26-containing formulations. The lower chamber was filled with a FBS-free culture medium. After 1 h of incubation at 37°C, the cells in the lower chamber were observed by optical microscopy. Scale bars, 100 μm.

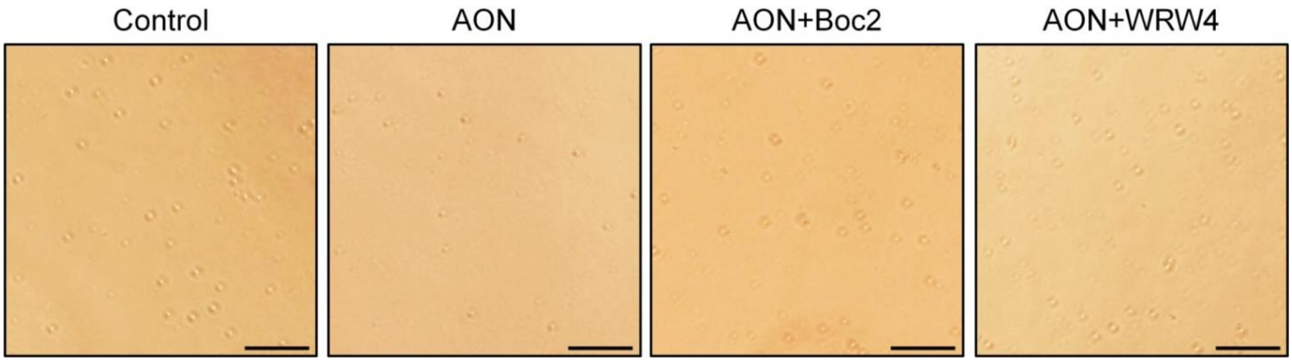


**Figure S28.** Microscopic images of neutrophils migrated to the lower chamber of the transwell system after treatment with different formulations. Neutrophils were separately exposed to vehicle (the control group), AON, AON and 1 μM Boc2, or AON and 10 μM WRW4 on the upper chamber of the transwell system. For AON, the dose was 1 μM of Ac2-26. The lower chamber was filled with a FBS-free culture medium. After 1 h of incubation at 37°C, the cells in the lower chamber were observed by optical microscopy. Scale bars, 100 μm.

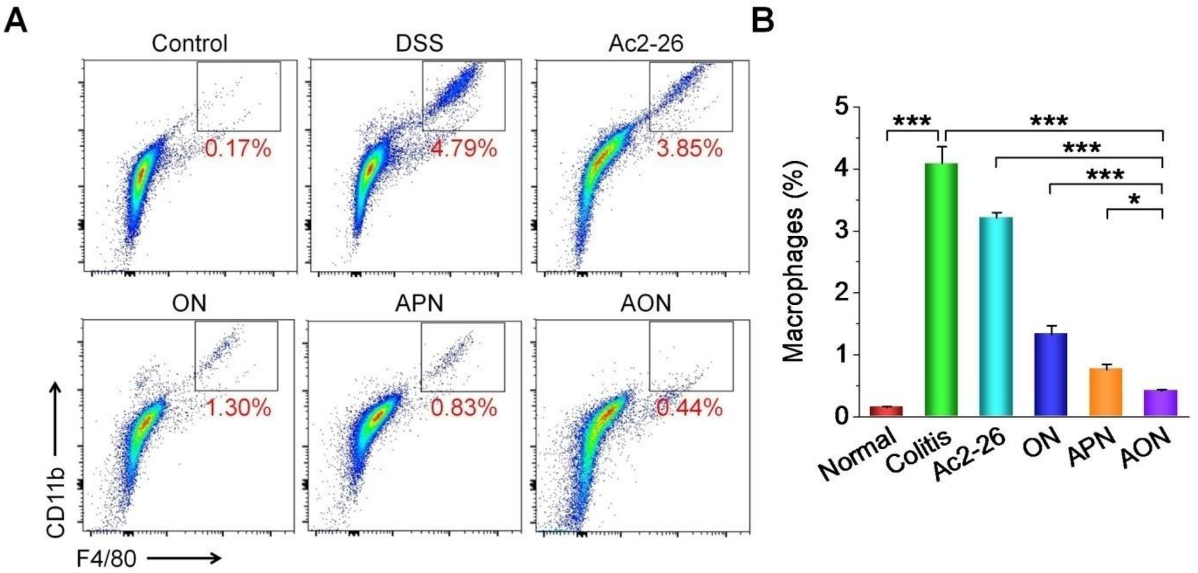


**Figure S29.** Flow cytometric analysis of macrophages in colonic tissues of mice with DSS-induced acute colitis. (A-B) Flow cytometric profiles (A) and quantitative analysis (B) showing total macrophage ($CD11b^+F4/80^+$) counts in single-cell suspensions derived from colonic tissues of mice with DSS-induced acute colitis after 7 days of treatment. Colitis mice received daily oral administration of saline, free Ac2-26, ON, APN, and AON, respectively, during 7 days of DSS treatment, while healthy mice in the normal group were not treated. The dose was 250 µg/kg of Ac2-26 for all Ac2-26-containing formations. Data are presented as mean ± SE ($n$ = 3). $^{*}P < 0.05$, $^{***}P < 0.001$.

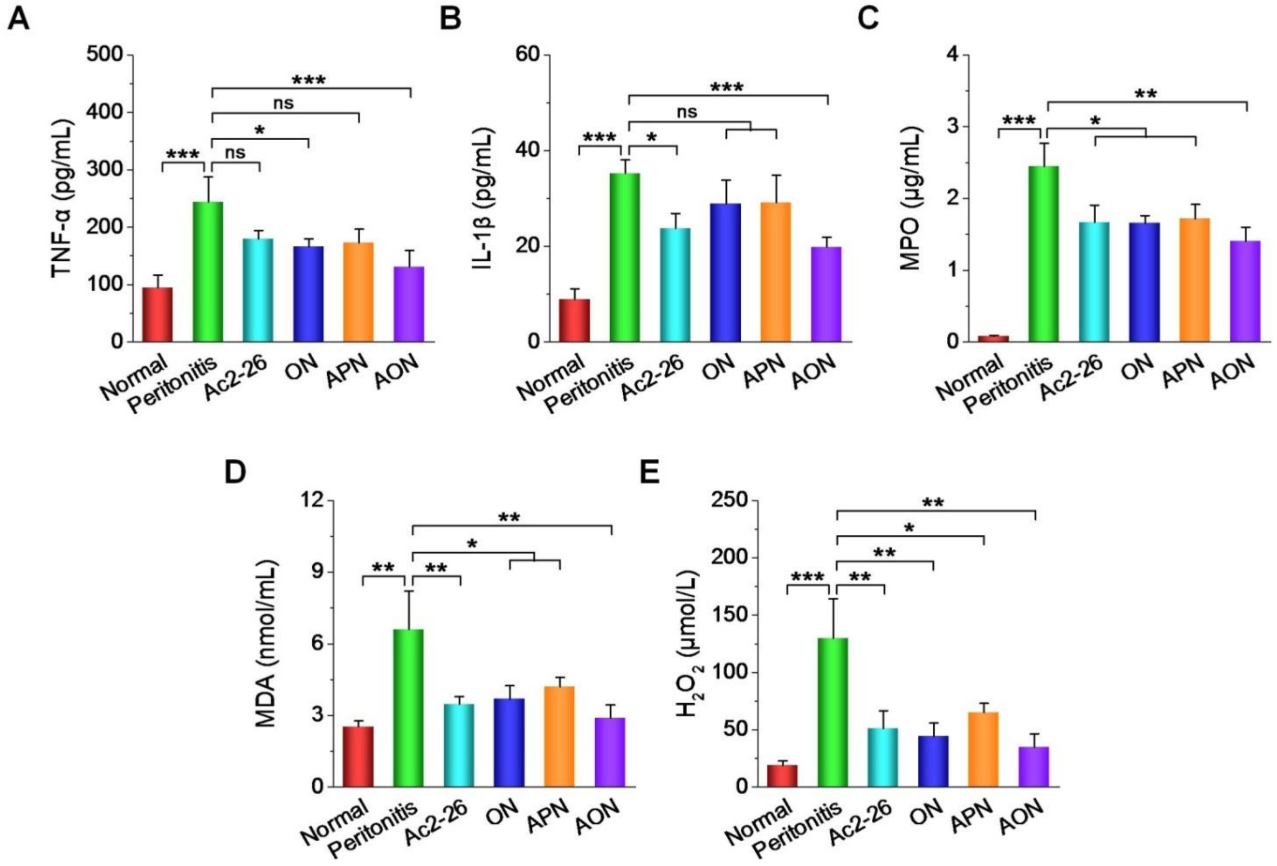


**Figure S30.** In vivo anti-inflammatory effects of AON in mice with zymosan-induced peritonitis in mice. (A-B) The concentrations of TNF-α (A) and IL-1β (B) in peritoneal lavage fluid. (C-E) The levels of MPO (C), MDA (D), and $H_2O_2$ (E) in peritoneal exudates. Mice were i.p. administered with 1 mg zymosan. At 1 h after zymosan injection, mice received i.p. injection of saline, free Ac2-26, ON, APN, or AON. For all Ac2-26 containing formulations, the dose was 500 ng Ac2-26 per mouse. After 6 h, peritoneal exudates were collected and the levels of various mediators were quantified using the corresponding assay kits. Data are presented as mean ± SE ($n$ = 6). $^{*}P < 0.05$, $^{**}P < 0.01$, $^{***}P < 0.001$; ns, no significance.

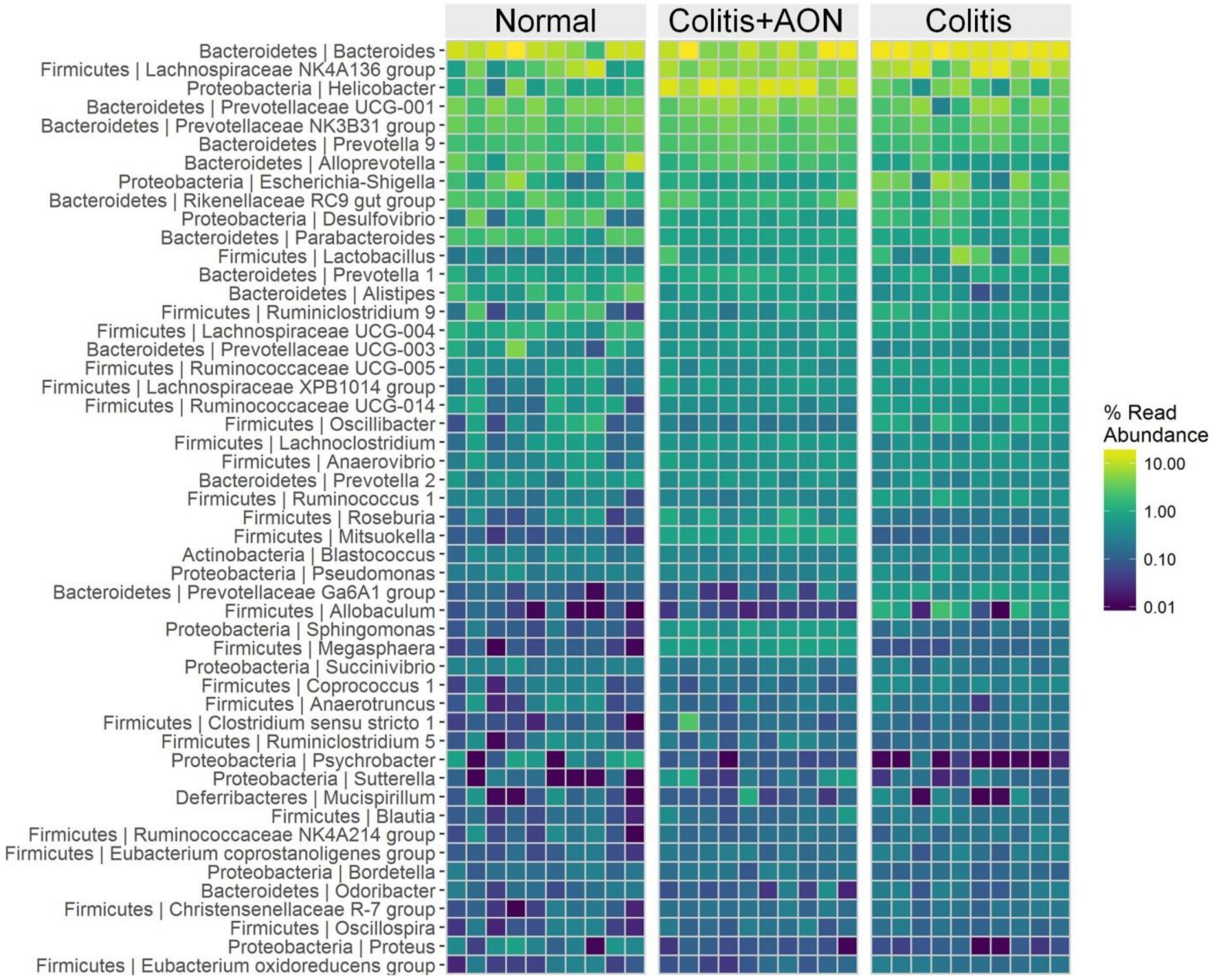


**Figure S31.** Heat map of bacterial taxon-based analysis at genus level in feces by 16S rRNA sequencing. Healthy mice in the normal group were not treated, while colitis mice were orally administered with saline or AON. For all diseased mice, daily administration was performed during 7 days of 3% DSS treatment. The dose was 250 µg/kg of Ac2-26 for the AON group. Fecal samples were collected, and total DNA was extracted for 16S rRNA gene sequence analysis. In each group, 10 mice were used.

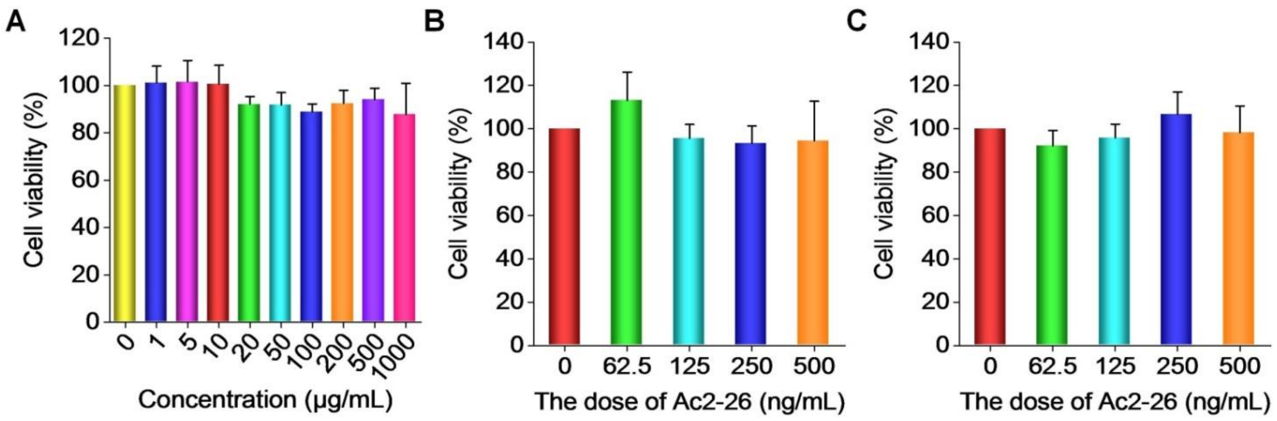


**Figure S32.** Cell viability of various nanoparticles in RAW264.7 macrophages. (A) ON. (B) AON. (C) APN. After 24 h of incubation with various doses of NPs, cell viability of RAW264.7 cells was quantified by MTT. Data are presented as mean ± SE (*n* = 5).

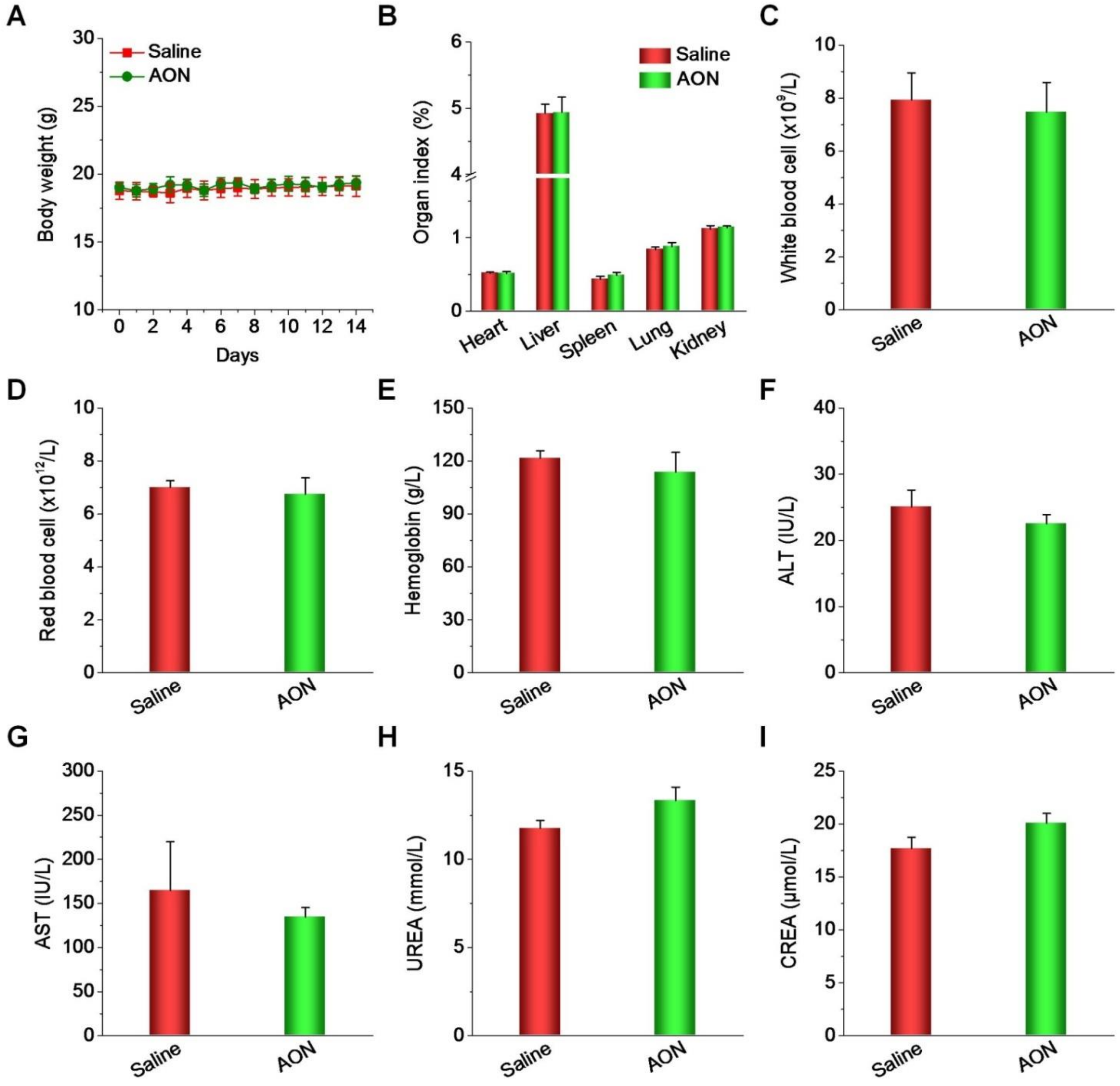


**Figure S33.** Safety evaluations of AON in mice after oral administration at 5 g/kg. (A) Changes in the body weight. (B) The organ index of major organs isolated at day 14 after treatment. (C-E) Typical hematological parameters including white blood cell (C), red blood cell (D), and hemoglobin (E). (F-I) Levels of ALT (F), AST (G), UREA (H), and CREA (I). Data are presented as mean ± SE ($n = 5$).

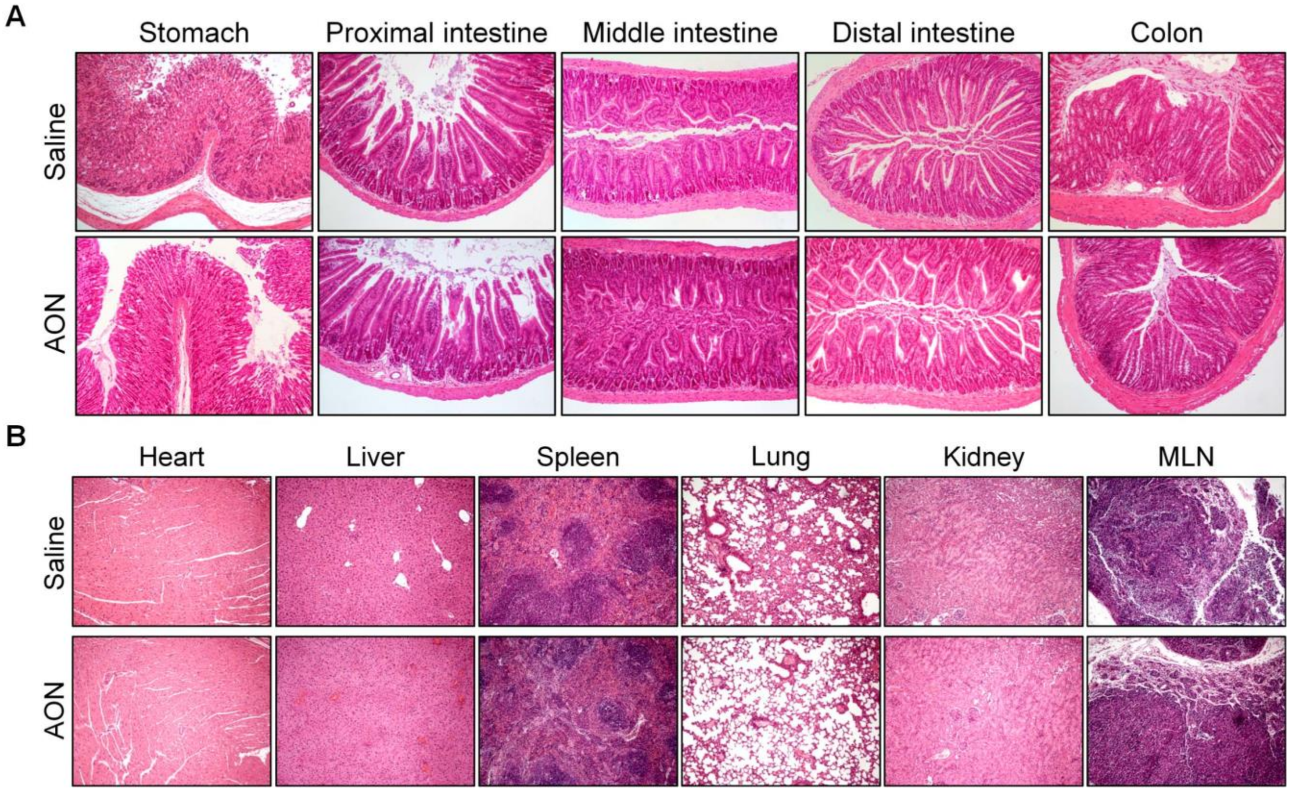


**Figure S34.** Histological evaluations of major organs from mice treated with a high dose of AON. (A-B) H&E-stained sections of gastrointestinal tissues (A) and major organs (B). The gastrointestinal tissues and organs were resected from mice at day 14 after oral administration of 5 g/kg of AON. After standard H&E staining, sections were observed by optical microscopy.
